# Fe–Fe bonding in the rhombic Fe_4_ cores of the Zintl clusters [Fe_4_E_18_]^4−^ (E = Sn and Pb)†

**DOI:** 10.1039/d4sc00165f

**Published:** 2024-02-28

**Authors:** Wei-Xing Chen, Zi-Sheng Li, Harry W. T. Morgan, Cong-Cong Shu, Zhong-Ming Sun, John E. McGrady

**Affiliations:** a State Key Laboratory of Elemento-Organic Chemistry, Tianjin Key Lab for Rare Earth Materials and Applications, School of Materials Science and Engineering, Nankai University Tianjin 300350 China sunlab@nankai.edu.cn; b Department of Chemistry, University of Oxford South Parks Road Oxford OX1 3QR UK john.mcgrady@chem.ox.ac.uk

## Abstract

We report here the synthesis and characterization of two endohedral Zintl-ion clusters, [Fe_4_Sn_18_]^4−^ and [Fe_4_Pb_18_]^4−^, which contain rhombic Fe_4_ cores. The Fe–Fe bond lengths are all below 2.5 Å, distinctly shorter than in the corresponding Cu clusters, indicating the presence of Fe–Fe bonding. Subtle differences in the structure of the Fe_4_ core between the two clusters suggest that the change in tetrel element causes a change in electronic ground state, with a very short Fe–Fe bond length of 2.328 Å present across the diagonal of the rhombus in the lead case.

## Abstract 2

We report here the synthesis and characterization of two endohedral Zintl-ion clusters, [Fe_4_Sn_18_]^4−^ and [Fe_4_Pb_18_]^4−^, which contain rhombic Fe_4_ cores.

## Graphical abstract

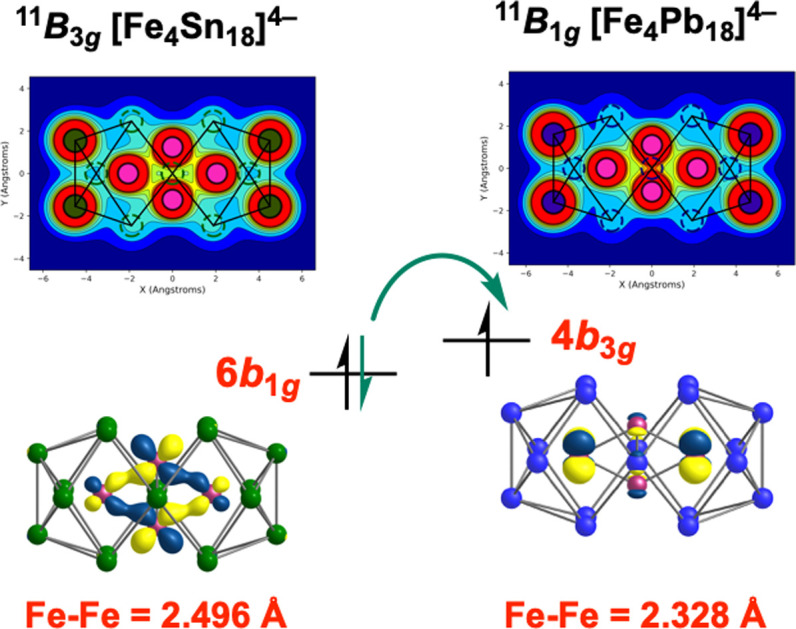

## 1 Introduction

Zintl clusters, and in particular those that contain encapsulated transition metals or f-elements, offer a fascinating insight into chemical bonding on the boundary between molecular systems and nanoparticles/nanoalloys.^1–6^ Some of the earliest examples, such as icosahedral [M@Pb_12_]^2−^,^7,8^ are relatively simple from an electronic perspective because the endohedral metal has a closed-shell (d^10^) configuration, and the core-like nature of the d orbitals limits the degree of interaction with the cage. The incorporation of earlier transition metals with fewer d electrons raises the possibility of paramagnetism, as is observed in [Mn@Pb_12_]^3−^ ((ref. 9) (a triplet)) and [Fe@Ge_10_]^3−^ ((ref. 10) (a doublet)).^11,12^ The relative destabilisation of the d orbitals also leads to stronger interactions with the cage (‘back-bonding’) which can, ultimately, drive structural changes, from the deltahedra that are characteristic of the electron deficient regime, to 3-connected ‘fullerene-like’ structures such as that of [Ru@Ge_12_]^3−^ that are the signature of more electron-rich cages.^13–16^

**Fig. 1:**
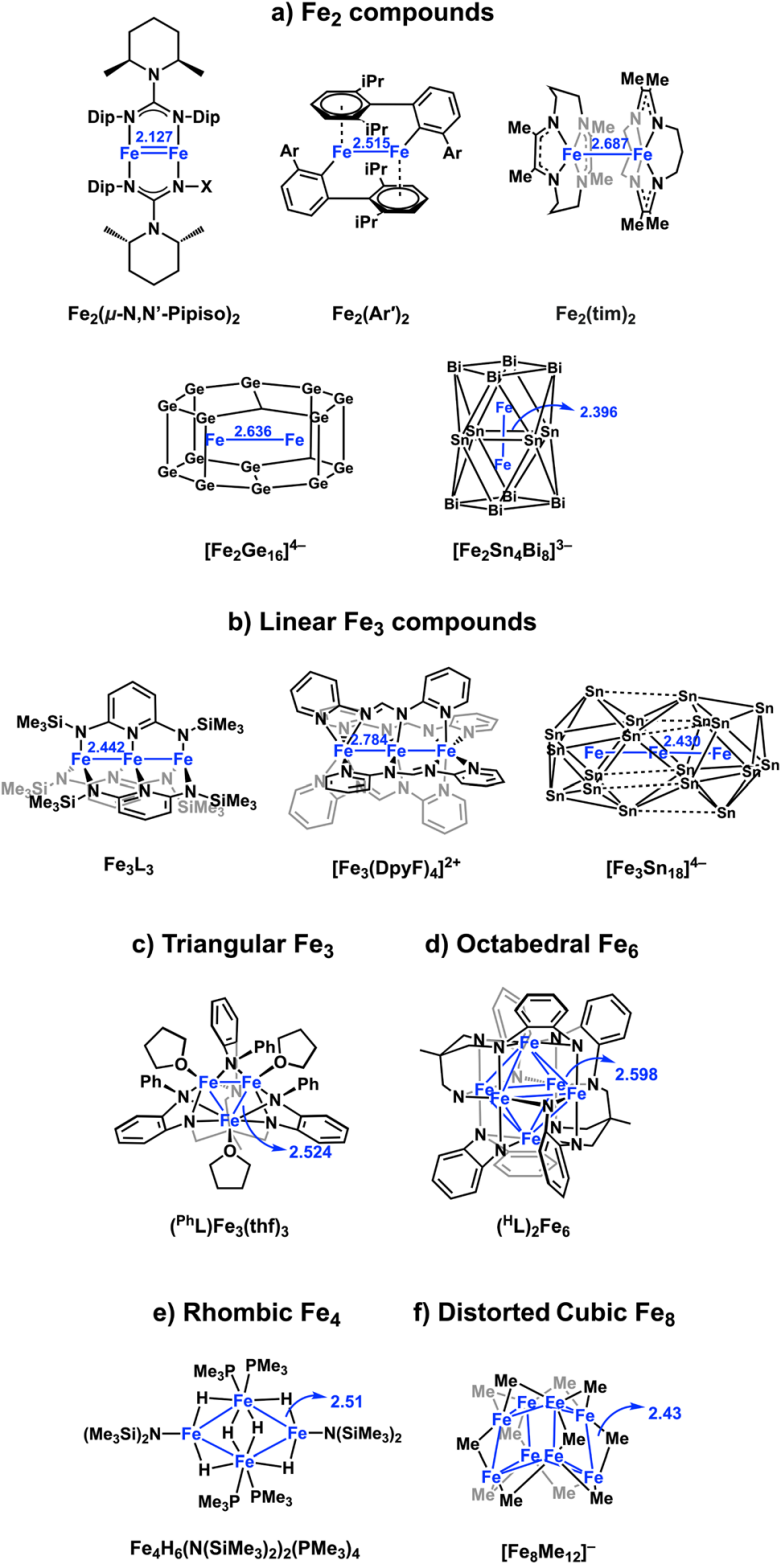
Structures of selected Fe–Fe bonded clusters in the literature, including (a) Fe_2_,^17,32–34^ (b) linear Fe_3_,^19–21^ (c) triangular Fe_3_,^22,23^ (d) octahedral Fe_6_,^24–26^ (e) rhombic Fe_4_,^35^ (f) distorted cubic Fe_8_.^28^

The extension of these principles to larger clusters containing two or more open-shell transition metal ions opens up a further range of possibilities. If the encapsulated metal ions are dilute, cooperative magnetic phenomena (ferro- and anti-ferromagnetism) may emerge, while a closer approach brings covalent metal–metal bonding into play. In the latter scenario, metal–metal and metal–cage bonding may be complementary: overlap between the metals will destabilise antibonding orbitals, potentially to the extent that electrons may be driven onto the cage. Opportunities to explore this landscape of possibilities have, up until now, been restricted by the relatively small number of well-characterized examples containing two or more endohedral metals. Amongst the very few Zintl-ion clusters with direct covalent metal–metal bonds, the Fe–Fe bond length in [Fe_2_Ge_16_]^4−^ (ref. 17) (Fig. 1(a)) is 2.636(3) Å whilst in [Fe_2_Sn_4_Bi_8_]^3−^,^18^ it is remarkably short, at 2.396(4) Å. Very recently, we have also reported the tri-iron cluster [Fe_3_Sn_18_]^4−^ (Fig. 1(b)) which contains a linear array of Fe centres, again with a very short Fe–Fe bond length of 2.4300(9) Å.^19^ These Zintl clusters are members of a wider family of iron clusters with direct covalent Fe–Fe bonds, which includes the Fe^1^Fe^1^ paddlewheel complex with ultra-short Fe–Fe bonds (2.127 Å), linear Fe_3_ chains (Fig. 1(b))^20,21^ and triangular Fe_3_ (ref. 22 and 23) and octahedral Fe_6_ units.^24–27^ Ohki has also reported a series of hydride bridged Fe_4_ and Fe_6_ clusters based on a rhombic Fe_4_ core, with Fe–Fe distances between 2.5 and 2.7 Å (Fig. 1(e)). Neidig and co-workers have also recently characterized the structure of the previously elusive ‘Kochi cluster’, a [Fe_8_Me_12_]^−^ (Fig. 1(f)), a distorted cube with a doublet ground state and averaged Fe–Fe bonds lengths of 2.43 Å.^28,29^ Paramagnetism is ubiquitous in the clusters shown in Fig. 1, and their electronic structure has typically been interpreted in terms of a delocalized manifold of Fe-based orbitals, occupied to maximise the multiplicity (‘Hund's coupling’).^30^

In a previous paper we have reported the structures of the copper-containing clusters, [Cu_4_Sn_18_]^4−^ and [Cu_4_Pb_18_]^4−^.^31^ We now extend that work to the earlier transition metals by reporting the properties of two new Zintl clusters, [Fe_4_Sn_18_]^4−^ and [Fe_4_Pb_18_]^4−^, both of which contain rhombic Fe_4_ cores, isostructural with the Cu analogues. The Fe–Fe separations (2.328 Å to 2.498 Å) are, however, shorter than those in [Cu_4_E_18_]^4−^ (2.52–2.55 Å), consistent with the presence of Fe–Fe bonding. The Fe_4_ cores are superficially very similar, but subtle differences suggest that the nature of the Fe–Fe bonding may be different in the Sn_18_ and Pb_18_ clusters. This observation motivates a detailed survey of the electronic structure using density functional theory, where we draw on comparisons both between the Sn and Pb species and also between the Fe and Cu analogues.

## 2 Results and discussion

### 2.1 Synthesis and properties of [Fe_4_E_18_]^4−^, E = Sn, Pb

**Fig. 2:**
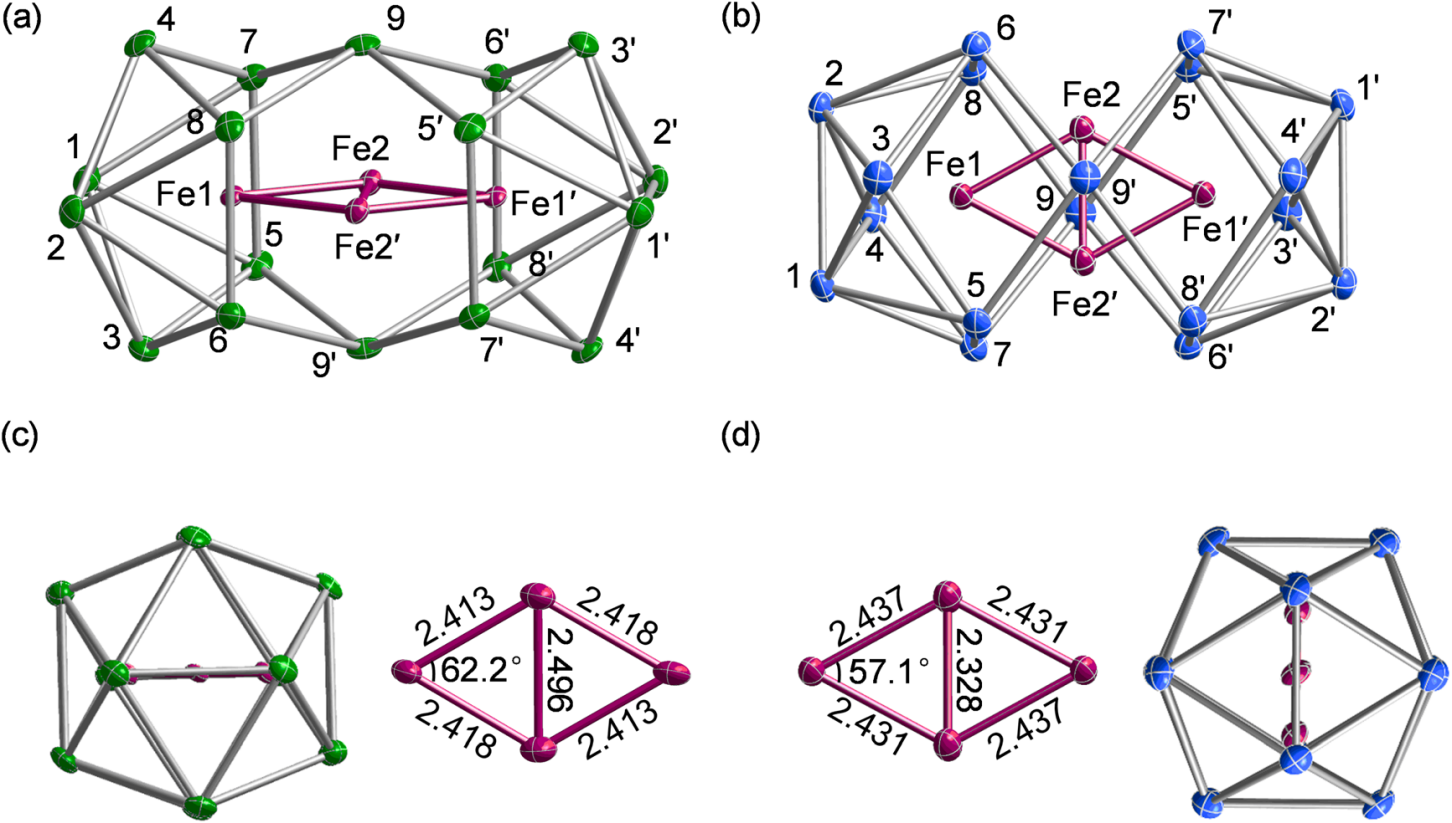
Molecular structures of the anionic components, [Fe_4_Sn_18_]^4−^ and [Fe_4_Pb_18_]^4−^. The two anions are isostructural: [Fe_4_Sn_18_]^4−^ is viewed along the *x* axis in (a) while [Fe_4_Pb_18_]^4−^ is viewed along *z* in (b). Fe–Fe bond lengths in the Fe_4_ rhombus are also shown in (c) and (d).

[K(2.2.2-crypt)]_4_[Fe_4_Sn_18_]·4Py (1) and [K(2.2.2-crypt)]_4_[Fe_4_Pb_18_]·4Py (2), were obtained from the reactions of ethylenediamine solutions of K_4_E_9_ with ferrocene in the presence of 2.2.2-crypt (further details are given in the experimental section). The reaction protocol differs from that used previously^19^ to synthesise the [Fe_3_Sn_18_]^4−^ cluster only in the source of Fe: ferrocene here but [K(thf)Fe(O^*t*^Bu)_3_]_2_ (thf = tetrahydrofuran). The two clusters, [Fe_3_Sn_18_]^4−^ and [Fe_4_Sn_18_]^4−^, appear not to interconvert, even in the presence of excess Fe. 1 crystallizes in the monoclinic space group *P*2_1_/*n* and contains a single anionic stannaspherene unit [Fe_4_Sn_18_]^4−^ along with four [K(2.2.2-crypt)]^+^ cations and four pyridine molecules in the asymmetric unit (Fig. 2(a)). The [Fe_4_Pb_18_]^4−^ unit 2 is isostructural with 1 (Fig. 2(b)), both having approximate D_2h_ symmetry, as do their copper analogues, [Cu_4_Sn_18_]^4−^ and [Cu_4_Pb_18_]^4−^.^31^ Two of the Fe atoms (Fe1 and Fe1′) are encapsulated in 8-vertex E_8_ polyhedra while Fe2 and Fe2′ sit in approximately trigonal prismatic sites either side of the waist of the cluster. Averaged bond lengths for all four clusters are collected together in Table 1 for comparison. The gross features of the E_18_ cage are largely unaffected by the change from Cu to Fe, with E–E bond lengths deviating by less than 0.05 Å in all cases. There are, however, subtle changes in M–M distances that reflect the emergence of metal–metal bonding in the iron clusters. As we have noted above, the d^10^ configuration of the Cu^+^ ions limits direct Cu–Cu bonding to the cuprophilic type, and so the short Cu1–Cu2 distances of ∼2.53 Å must reflect, at least to some extent, the constraints imposed by the E_18_ framework. Irrespective of whether the main-group element is Sn or Pb, the Fe1–Fe2 bond lengths are shorter than the Cu1–Cu2 analogues by approximately 0.1 Å. Differences in the M2–M2′ distances are, in contrast, rather more variable: in the Sn clusters, the Fe2–Fe2′ is shorter than Cu2–Cu2′ by 0.135 Å, but this difference increases to 0.233 Å for the Pb pair. Indeed the Fe2–Fe2′ distance of 2.328 Å in [Fe_4_Pb_18_]^4−^ is comparable to the shortest Fe–Fe bonds shown in Fig. 1. The correlation of bond length and bond strength is not always straightforward in clusters with bridging ligands, but we note that for a given oxidation state the radius of Fe is intrinsically larger than that of Cu, so the shorter Fe–Fe distances within a constant E_18_ framework are a strong *a priori* indication of direct Fe–Fe bonding. Moreover, the wider variation in the M2–M2′ bond lengths is probably a reflection of the fact that these atoms are not fully encapsulated by the cluster, and are therefore more free to move in response to subtle changes in Fe–Fe bonding. We explore the origins of these structural differences as part of a detailed analysis of electronic structure presented in the following sections.

**Table 1:** Selected bond lengths from crystallographic and DFT-optimized structures (M06-L functional) for the [M_4_E_18_]^4−^ family (all distances in Å)

		*E*_rel_/eV	M1–M2	M2–M2′	M1–E3	M2–E9	E2–E6	E5–E9′	E7–E8′	Ref
[Fe_4_Sn_18_]^4−^	X-ray (100 K)		2.413	2.496	2.828	2.739	3.142	3.225	4.898	This work
	DFT (^9^A_g_)	0.59	2.41	2.47	2.90	2.75	3.17	3.18	4.89	
	DFT (^11^B_1g_)	0.0	2.42	2.31	2.90	2.79	3.20	3.19	4.88	
	DFT (^11^B_3g_)	0.05	2.41	2.52	2.88	2.75	3.17	3.24	4.86	
	DFT (^13^B_2g_)	0.25	2.43	2.63	2.94	2.75	3.14	3.27	5.06	
[Fe_4_Pb_18_]^4−^	X-ray (100 K)		2.437	2.328	2.974	2.892	3.247	3.287	4.941	This work
	DFT (^9^B_1u_)	0.79	2.41	2.46	2.99	2.90	3.32	3.37	4.99	
	DFT (^11^B_1g_)	0.0	2.44	2.28	3.03	2.90	3.35	3.32	4.99	
	DFT (^11^B_3g_)	0.18	2.43	2.48	3.00	2.86	3.32	3.36	4.98	
	DFT (^13^B_3g_)	0.18	2.46	2.35	3.01	2.86	3.34	3.37	5.05	
[Cu_4_Sn_18_]^4−^	X-ray (100 K)		2.525	2.631	2.842	2.760	3.108	3.219	4.915	31
	DFT (^1^A_g_)		2.52	2.58	2.86	2.76	3.14	3.24	4.91	
[Cu_4_Pb_18_]^4−^	X-ray (100 K)		2.547	2.561	2.935	2.825	3.224	3.299	5.012	31
	DFT (^1^A_g_)		2.55	2.52	2.99	2.88	3.30	3.35	5.02	

**Fig. 3:**
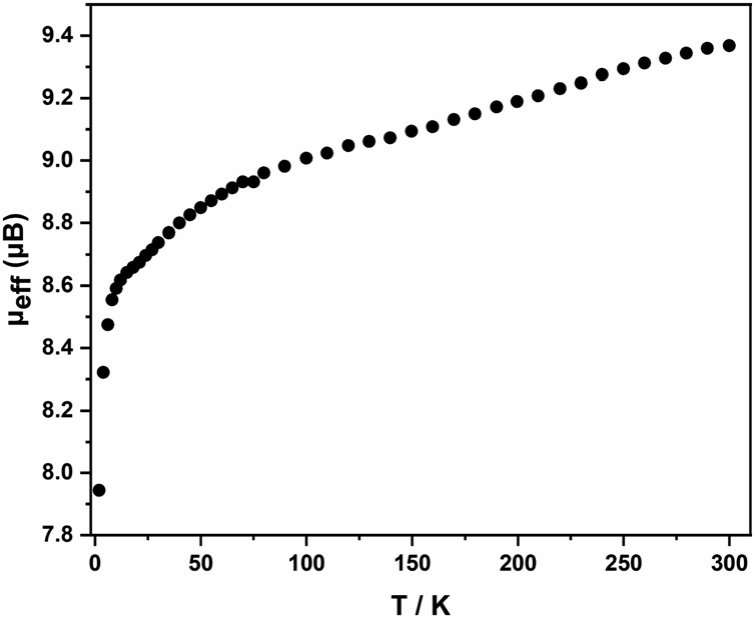
Effective magnetic moment, *μ*_eff_, as a function of temperature for 1.

Electrospray ionization mass spectra (ESI-MS) of freshly prepared samples of 1 and 2, measured in negative-ion mode, are shown in Fig. S9–15.† For 1, the spectrum shows an intense feature due to the dianion [Fe_4_Sn_18_]^2−^, as well as smaller features due to the monoanion, [Fe_4_Sn_18_]^−^ and the dianion paired with a single [K(2.2.2-crypt)]^+^ cation, [K(2.2.2-crypt)Fe_4_Sn_18_]^−^. The corresponding spectrum for 2, in contrast, shows a single dominant peak due to the decomposition product [FePb_12_]^−^, most likely an endohedrally encapsulated icosahedron, similar in structure to the known [Mn@Pb_12_]^3−^ cluster. The only possible trace of the intact cluster is a very small peak, just above the baseline, centreed at *m/z* = 1977: it is clear that the parent cluster does not survive in any significant quantities under ESI-MS conditions. The magnetic susceptibility of 1 was measured between 2 and 300 K using a superconducting quantum interference device (SQUID), Fig. 3. The measured magnetic moments (*μ*_eff_) decreases with decreasing temperature from a value of 9.37 *μ*_B_ (*χ*_M_*T* = 10.97 cm^3^ K mol^−1^) at 300 K to ∼9 *μ*_B_ at 100 K, before dropping to 8.6 *μ*_*B*_ at 10 K, and, finally, to much lower values below this temperature. The drop off below 10 K may be the result of zero-field splitting and/or weak antiferromagnetic coupling between cluster units. Limiting spin-only (*g* = 2) values of *μ*_eff_ for *S* = 4, *S* = 5 and *S* = 6 are 8.94 *μ*_B_, 10.95 *μ*_B_ and 12.96 *μ*_B_, respectively, and the absence of a plateau at 300 K suggests that the ground spin state is not isolated. Despite multiple attempts on freshly prepared samples, we were not able to make reproducible magnetic measurements on the lead analogue, 2: this probably reflect the difficulties in separating crystals of 2 from other side products of the reaction while preparing the sample for magnetic measurements and/or rapid decomposition of the sample, as is apparent in the ESI-MS experiment.

### 2.2 Electronic structure

The electronic structure of the two Cu clusters, [Cu_4_Sn_18_]^4−^ and [Cu_4_Pb_18_]^4−^, was the subject of a previous study^31^ where we concluded that the ground states were singlets, with Cu^+^ ions in a d^10^ configuration and no direct Cu–Cu interactions. In contrast, the open-shell Fe centres generate several closely-spaced electronic states that differ both in multiplicity and spatial symmetry, and the energies of the lowest-lying states with 2*S* + 1 = 9, 11 and 13, calculated with the M06-L functional, are summarized in Table 1. Amongst these, the two lowest share a common spin, *S* = 5, but have different spatial symmetries, ^11^B_1g_ and ^11^B_3g_: the lowest-lying state with *S* = 4 lies more than 0.5 eV higher in energy. The measured magnetic moment of 9.37 *μ*_B_ for [Fe_4_Sn_18_]^4−^ at 300 K lies considerably below the spin-only value for *S* = 5 (10.95 *μ*_B_), and in fact is more consistent with a ground-state with *S* = 4. The apparent discrepancy between computed and experimental values may be a consequence of the well-known sensitivity of ground-state multiplicity to choice of density functional or, alternatively, may indicate the presence of diamagnetic impurities in the sample that serve to depress the measured moment. Of the two states with 2*S* + 1 = 11, ^11^B_1g_ is the most stable for both [Fe_4_Pb_18_]^4−^ and [Fe_4_Sn_18_]^4−^, although the energetic difference is too small to allow a confident assignment of the ground state, particularly in the Sn cluster where the two differ by only 0.05 eV. However, a comparison of optimized and measured bond lengths offers important clues to the identity of the electronic ground states. The optimised Fe2–Fe2′ bond is short in the ^11^B_1g_ state (2.31 Å and 2.28 Å for Sn and Pb, respectively), while the Fe1–Fe2 bond is relatively long (2.42 Å and 2.44 Å for Sn and Pb, respectively). In the ^11^B_3g_ state, the opposite pattern is found: the Fe2–Fe2′ bond is long (2.52 Å for Sn and 2.48 Å for Pb) while the Fe1–Fe2 bond is somewhat shorter (2.41 Å for Sn and 2.43 Å for Pb). When compared to experiment, it is clear that the ^11^B_3g_ state offers a much better match to the X-ray structure of [Fe_4_Sn_18_]^4−^ (Fe1–Fe2 = 2.413 Å, Fe2–Fe2′ = 2.496 Å) while the ^11^B_1g_ state offers a better match to the X-ray structure of [Fe_4_Pb_18_]^4−^ (Fe1–Fe2 = 2.437 Å, Fe2–Fe2′ = 2.328 Å). On that basis, and despite the fact that the ^11^B_3g_ state is 0.05 eV less stable than ^11^B_1g_, we believe that former is the best candidate for the electronic ground state of the Sn cluster.

**Fig. 4:**
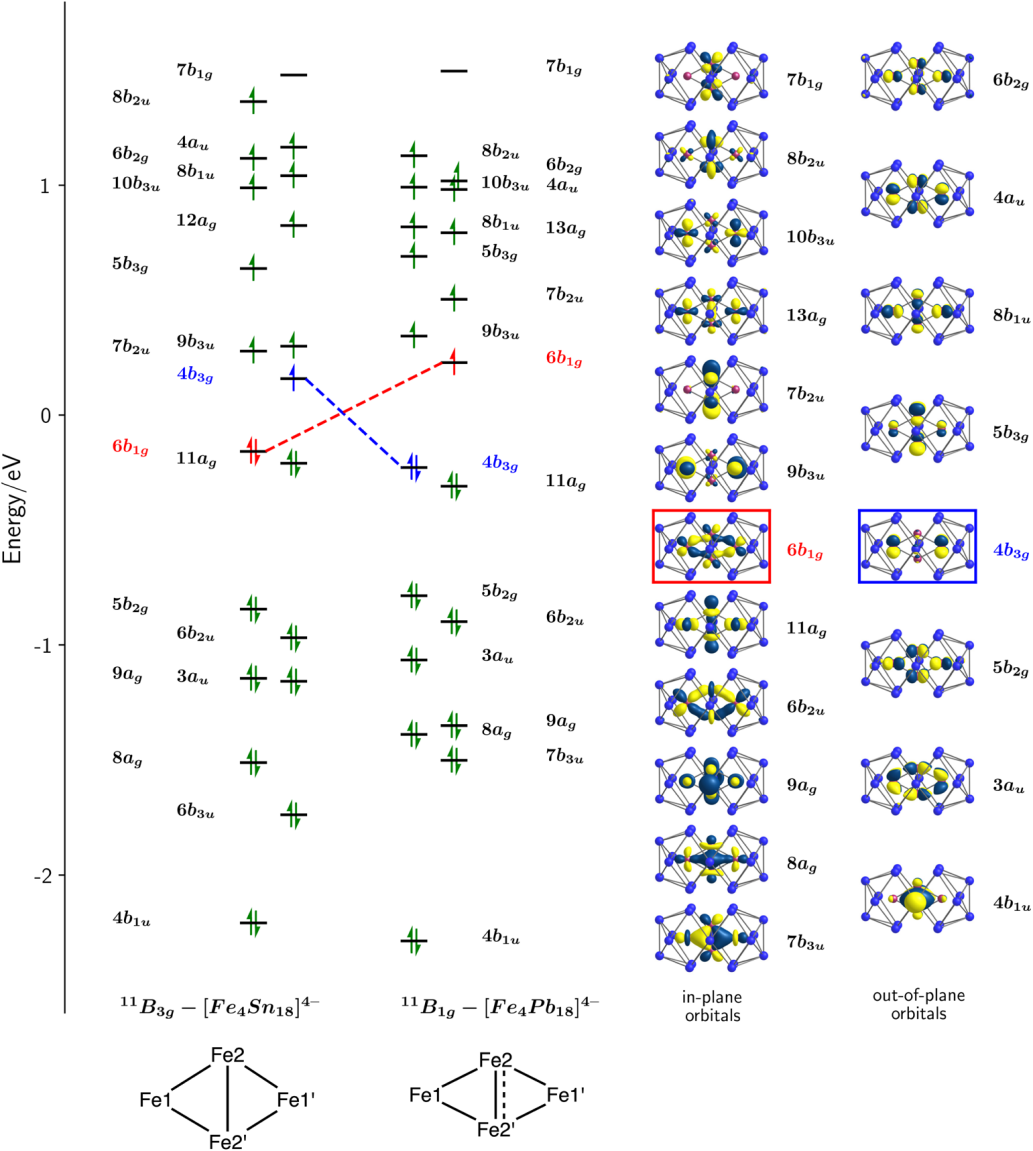
Kohn–Sham molecular orbitals for the ^11^B_3g_ of [Fe_4_Sn_18_]^4−^ and the ^11^B_1g_ state of [Fe_4_Pb_18_]^4−^. The eigenvalues correspond to the spin-β manifold in both cases, and they are shifted such that *E* = 0 is defined as the mid-point between the eigenvalues of HOMO and LUMO. The orbitals highlighted in red and blue are those whose occupations differ in the ^11^B_3g_ and ^11^B_1g_ states. The isosurfaces shown correspond to the spin-β set of [Fe_4_Pb_18_]^4−^.

A schematic representation of the Kohn–Sham orbitals for [Fe_4_Sn_18_]^4−^ and [Fe_4_Pb_18_]^4−^ in their respective ^11^B_3g_ and ^11^B_1g_ states is shown in Fig. 4, and a full analysis of the orbitals, projected densities of states (PDOS), and overlap-projected densities of states (OPDOS) is presented in the ESI.† The interpretation of the electronic structure is a complex challenge due to the open-shell character, and a consideration from a symmetry-based perspective is a useful starting point. The rhombic Fe_4_^4+^ unit has a total of 4 × 5 = 20 linear combinations of Fe 3d orbitals, which can be separated into in-plane (a_g_, b_1g_, b_2u_, b_3u_ symmetries) and out-of-plane (a_u_, b_1u_, b_2g_ and b_3g_) subsets. The in-plane set support Fe–Fe interactions of local σ and π_ip_ symmetry, while the out-of-plane set have local Fe–Fe π_op_ and δ symmetry.§§ip and op denote in-plane and out-of-plane, respectively. The formal oxidation state, and therefore the number of electrons occupying the Fe 3d manifold, is not straightfoward due to the multiple redox states available to Fe. However, the structures of the E_18_ units in 1 and 2 and also the Sn/Pb densities of states (ESI, Fig. S22†) are striking similar to those in the [Cu_4_E_18_]^4−^ cases, where the charge state of the E_18_ unit was assigned as 8-.^31^ The relationship between structure and charge in the family of 18-vertex tetrel clusters has been discussed by us and others in the recent literature,^19,36–40^ and we provide further justification for the assignment of an 8- charge state in the ESI.† Assuming the same 8- charge for the E_18_ unit in [Fe_4_E_18_]^4−^, we then have a net 4+ charge for the Fe_4_ unit and hence 28 valence electrons in the Fe 3d manifold. Distributing these 28 valence electrons across the 20 orbitals generates a maximum possible spin of *S* = 6 (the ^13^B_2g_ state identified in Table 1). In the ground states of the two clusters with 2*S* + 1 = 11, (^11^B_1g_ and ^11^B_3g_) the highest of these 20 linear combinations, 7b_1g_, is vacant in both spin-α and spin-β manifolds, leaving 9 doubly-occupied and 10 singly-occupied orbitals. The doubly-occupied levels are largely Fe1–Fe2 and Fe2–Fe2′ bonding while the singly-occupied levels are largely antibonding, giving rise to net Fe–Fe bonding, in contrast to the Cu analogues where all bonding and antibonding combinations are filled (see the discussion of the PDOS and OPDOS in the ESI† for more details). We can identify three distinct bonding/antibonding orbital pairs that make substantial contributions to the Fe2–Fe2′ bonding in both the ^11^B_1g_ and ^11^B_3g_ states: the 8a_g_^2^8b_2u_^1^ configuration (Fe2–Fe2′*σ*), the 4b_1u_^2^5b_3g_^1^ configuration (π_op_) and the 7b_3u_^2^7b_1g_^0^ configuration (π_ip_). The key difference between the ^11^B_1g_ and ^11^B_3g_ states lies in the reversal of the 6b_1g_ and 4b_3g_ orbitals (highlighted in red and blue, respectively, in Fig. 4) the first of which has significant Fe2–Fe2′ π_ip_* character while the second is localised largely on the Fe1 centres with little amplitude on the central Fe2–Fe2′ unit. The in-plane component of the Fe2–Fe2′ bond is therefore stronger in the ^11^B_1g_ state, leading to compressed bond in [Fe_4_Pb_18_]^4−^. The differences between Cu and Fe, and between [Fe_4_Sn_18_]^4−^ and [Fe_4_Pb_18_]^4−^, are captured in the Mayer bond orders (MBO),^41^ delocalization indices (DI)^42^ and bond critical point ellipticities (*ε*) shown in the ESI, Table S6.† The Mayer bond orders of 0.51 and 0.91 for the Fe2–Fe2′ bonds of [Fe_4_Sn_18_]^4−^ and [Fe_4_Pb_18_]^4−^ in their respective ground states are a clear indication of the stronger bond in the latter while the Fe1–Fe2 bonds show the opposite trend (0.85 and 0.69). The corresponding values are, on average, less than 0.2 for the Cu clusters, where formal Cu–Cu bond orders are zero. The ellipticity at the critical point, *ε*, provides a measure of the balance between σ, π_ip_ and π_op_ character: values close to 0 are indicative of a cylindrically symmetric environment while larger values indicate an anisotropic π component to the bond. The values of *ε* ∼0.2 for the Cu clusters again establish a useful reference point. The ellipticity of 0.46 the Fe2–Fe2′ bond of [Fe_4_Pb_18_]^4−^ in its ground state is much lower than the value of 2.11 for the corresponding bond in the ^11^B_3g_ ground state of [Fe_4_Sn_18_]^4−^, the high value in the latter case reflecting the substantial contribution of the π_op_ bonding mediated by the 4b_1u_/5b_3g_ pair. In the ^11^B_1g_ ground state of [Fe_4_Pb_18_]^4−^, this out-of-plane π component is supplemented by an additional π_ip_ component resulting from the removal of one electron from the Fe–Fe π antibonding 4b_3g_ orbital. In summary, our analysis of the electronic structure reveals that (a) There is significant Fe–Fe covalent bonding in both 1 and 2, in contrast to the situation in the Cu analogues where bonding is absent and (b) [Fe_4_Sn_18_]^4−^ and [Fe_4_Pb_18_]^4−^ have different electronic configurations, leading to a marked change in the structure of the Fe_4_ rhombus with a significantly contracted Fe2–Fe2′ bond in the latter.

## 3 Summary and conclusions

In this paper we have reported the synthesis and characterization of two new Fe-based Zintl clusters, [Fe_4_Sn_18_]^4−^, [Fe_4_Pb_18_]^4−^, both of which have rhombic Fe_4_ cores. The clusters are isostructutal with the previously-reported Cu analogues, but the Fe–Fe bonds, all of which are below 2.5 Å, are substantially shorter than the corresponding Cu–Cu distances, indicating the presence of Fe–Fe bonding. There are also significant differences between the Fe_4_ cores in the Sn and Pb clusters, with the Fe2–Fe2′ bond across the diagonal of the rhombus being notably shorter in the latter (2.328 Å *vs.* 2.498 Å). Magnetic susceptibility measurements for [Fe_4_Sn_18_]^4−^ indicate a high-spin ground state, with limiting values of *μ*_eff_ consistent with either *S* = 5 or *S* = 6. A survey of the potential energy surface using density functional theory identifies two almost degenerate states with *S* = 5 as the most stable (M06-L functional), with very different Fe2–Fe2′ bond lengths that map onto the differences seen in the crystallography. On that basis we believe that the two clusters adopt ground states with the same multiplicity but different spatial symmetry, with Fe–Fe π bonding more developed in the [Fe_4_Pb_18_]^4−^ case.

## 4 Materials and methods

### 4.1 Materials and reagents

All manipulations and reactions were performed under a nitrogen atmosphere using standard Schlenk or glovebox techniques. Ethylenediamine (en) (Aldrich, 99%) and DMF (Aldrich, 99.8%) were freshly distilled by CaH_2_ prior to use, and stored in N_2_ prior to use. Tol (Aldrich, 99.8%) was distilled from sodium/benzophenone under nitrogen and stored under nitrogen. 2.2.2-crypt (4,7,13,16,21,24-Hexaoxa-1,10-diazabicyclo (8.8.8) hexacosane, purchased from Sigma-Aldirich, 98%) was dried in vacuum for one day prior to use. FeCp_2_ was purchased from Energy Chemical (China). K_4_E_9_ was synthesized by heating a stoichiometric mixture of the elements (K: +99%, Sn: 99.99% and Pb: 99.99% all from Aladdin) at 850 °C for 36 h in a niobium tube.

### 4.2 Synthesis of 1 and 2

#### 4.2.1 [K(2.2.2-crypt)]_4_[Fe_4_Sn_18_]·4Py(1)

In a 10 mL vial, K_4_Sn_9_ (122 mg, 0.1 mmol) and 2.2.2-crypt (115 mg, 0.3 mmol) were dissolved in en (*ca.* 3 mL) and stirred for 30 min, resulting a dark brown solution. Then FeCp_2_ (30 mg, 0.161 mmol) was dispersed in toluene (0.5 mL), producing a light pink suspension, and then added dropwise to the above mixture. The mixture was stirred for 3 h at room temperature yielding a brown solution. All volatiles were removed at 60 °C under vacuum to obtain a black solid which was dissolved in 3 mL pyridine. The resulting green-black solution was stirred for 2 h at room temperature and filtered with glass wool. The filtrate was layered with 3 mL toluene. Block-shaped crystals of 1 were obtained (20% yield based on the used precursor K_4_Sn_9_) after six weeks.

#### 4.2.2 [K(2.2.2-crypt)]_4_[Fe_4_Pb_18_]·4Py(2)

In a 10 mL vial, K_4_Pb_9_ (120 mg, 0.059 mmol), 2.2.2-crypt (88 mg, 0.234 mmol) and FeCp_2_ (22 mg, 0.118 mmol) were dissolved in 3 mL en yielding a black solution. The mixture was stirred for 3 h at room temperature yielding a brown solution. All volatiles were removed at 60 °C under vacuum to obtain a dark-brown solid which was dissolved in 3 mL pyridine. The resulting green-black solution was stirred for 1 h at room temperature and filtered with glass wool. The filtrate was layered with 3 mL toluene. Black rod-like crystals of 2 were obtained (13% yield based on the used precursor K_4_Pb_9_) after one week.

### 4.3 Experimental analysis

#### 4.3.1 X-ray diffraction

Suitable single crystals were selected for X-ray diffraction analyses. Crystallographic data were collected on Rigaku XtalAB Pro MM007 DW diffractometer with graphite monochromated Cu K*α* radiation (*λ* = 1.54184 Å).

#### 4.3.2 Electrospray ionization mass spectrometry (ESI-MS) investigations

Negative ion mode ESI-MS of the DMF solutions of the single crystal and reaction solution were measured on an LTQ linear ion trap spectrometer by Agilent Technologies ESI-TOF-MS (6230). The spray voltage was 5.48 kV and the capillary temperature was kept at 300 °C. The capillary voltage was 30 V. The samples were made up inside a glovebox under a nitrogen atmosphere and rapidly transferred to the spectrometer in an airtight syringe by direct infusion with a Harvard syringe pump at 0.2 mL min^−1^.

#### 4.3.3 Energy dispersive X-ray (EDX) spectroscopic analysis

EDX analysis on the title clusters were performed using a scanning electron microscope (FE-SEM, JEOL JSM-7800F, Japan). Data acquisition was performed with an acceleration voltage of 15 kV and an accumulation time of 60 s.

#### 4.3.4 Superconducting quantum interference devices (SQUID)

Magnetic measurements were performed using a Quantum Design SQUID MPMS-3 magnetometer in the 2.0 to 300 K temperature range with an applied field of 1000 Oe.

### 4.4 Computational details

All calculations are performed using density functional theory as implemented in the ADF 2021.104 package.^43^ The local functional of Minnesota 06 family (M06-L) functionals^44,45^ was used and triple-zeta basis sets included with two polarization functions are used for all atoms.^46^ All electrons are treated as valence in the calculations. Relativistic effect was considered with The Zeroth-Order Regular Approximation (ZORA).^47^ The numerical quality was set to ‘verygood’.^48^ A Conductor-like Screening Model (COSMO) with dielectric constant of 78.39 was used to simulate the confining environment of the ionic lattice.^49^ Optimized structures were confirmed to be minima through the absence of imaginary frequencies.^50^ The sensitivity of the results to choice of functional was also explored by repeating the calculations using the PBE and PBE0 functionals.^51,52^

## Data availability

Yes, the original data that supports the finding in the article are available in the ESI† and CCDC databases.

## Author contributions

W.-X. C. and C.-C. S. performed the synthesis and characterisation, Z. L. and H. W. T. M. performed the computational analysis. Z.-M. S. and J. E. M. conceived the project and supervised the experimental and computational aspects of the research, respectively. All authors contributed to the preparation of the manuscript.

## Conflicts of interest

There are no conflicts to declare.

## Supplementary Material

SC-015-D4SC00165F-s001

SC-015-D4SC00165F-s002
