# Supplementary material for: Fe–Fe bonding in the rhombic Fe4 cores of the Zintl clusters [Fe4E18]4− (E = Sn and Pb)
Source: Chem Sci. 2024 Feb 28;15(13):4981–8. doi: 10.1039/d4sc00165f (PMC10967007; doi:10.1039/d4sc00165f)
Supplement: SC-015-D4SC00165F-s001 [file SC-015-D4SC00165F-s001.pdf]

Electronic Supplementary Material (ESI) for Chemical Science.
This journal is © The Royal Society of Chemistry 2024

Supplementary Information for

Isomerism of the Rhombic Fe_4 core in the Zintl Clusters $[Fe_4E_{18}]^{4-}$ (E = Sn and Pb)

Table of Contents

1. Experimental Procedures S2
2. Crystallographic details S3
3. ESI-MS Studies S10
4. Energy Dispersive X-ray (EDX) Spectroscopic Analysis S13
5. Superconducting Quantum Interference Devices(SQUID) S15
6. Computational Details S16
7. References S39

1. Experimental Procedures

Synthesis of **[K(2,2,2-crypt)]$_4$[Fe$_4$Sn$_{18}$]·4DMF (1-DMF)**

In a 10 mL vial, K_4Sn_9 (122 mg, 0.1 mmol) and 2.2.2-crypt (115mg, 0.3 mmol) were dissolved in en (ca. 3 mL) and stirred for 30 min, resulting a dark brown solution. Then $FeCp_2$ (30 mg, 0.160 mmol) was dispersed in toluene (0.5 mL), producing a light pink suspension, and then added dropwise to the above mixture. The mixture was stirred for 3 h at room temperature yielding a brown solution. All volatiles were removed at 60°C under vacuum to obtain black solid which was dissolved in 3mL DMF. The resulting green-black solution was stirred for 1 h at room temperature and filtered with glass wool. The filtrate was layered with 3 mL toluene. A large number of brown plate-like crystals of **1-DMF** were obtained (17% yield based on the used precursor K_4Sn_9) after one week.

2. Crystallographic details

The structure of crystals **1**, **1-DMF** and **2** were solved using direct methods and then refined using SHELXL-2014 and Olex2[1-3] to convergence, in which all the non-hydrogen atoms were refined anisotropically during the final cycles. All hydrogen atoms of the organic molecule were placed by geometrical considerations and were added to the structure factor calculation. We used the PLATON SQUEEZE procedure[4] to remove the solvent molecules which could not be modeled properly. A summary of the crystallographic data for the title compounds was listed in table S1. CCDC entries 2206397 (**1**), 2170117 (**1-DMF**), and 2101583 (**2**) are included in the supplementary crystallographic data for this paper. These data can be obtained free of charge from The Cambridge Crystallographic Data Centre (www.ccdc.cam.ac.uk/data_request/cif).

Table S1. X-ray measurements and structure solutions of **1**, **1-DMF**, and **2**.

Compound	**1**	**1-DMF**	**2**
CCDC number	2206397	2170117	2101583
Empirical formula	$C_{92}H_{164}Fe_4K_4N_{12}O_{24}Sn_{18}$	$C_{42}H_{86}Fe_2K_2N_6O_{14}Sn_9$	$C_{46}H_{82}N_6O_{12}K_2Fe_2Pb_9$
Formula weight	4338.56	2157.27	2965.78
Temperature/K	100.01(11)	129.99(10)	100.00(10)
Crystal system	monoclinic	triclinic	monoclinic
Space group	$P2_1/n$	P-1	$P2_1/n$
a /Å	14.30740(10)	15.7369(2)	14.3298(1)
b /Å	27.4645(2)	15.9735(3)	27.6726(2)
c /Å	18.80560(10)	16.6129(2)	18.9178(2)
α/°	90	107.7610(14)	90
β/°	110.6780(10)	97.0389(12)	110.718(1)
γ/°	90	112.8799(15)	90
V /Å^3	6913.54(9)	3522.99(10)	7016.60(11)
Z	2	2	4
ρ_{calc} /g·cm^{-3}	2.084	2.034	2.808
$\mu(Cu_{K\alpha})$ /mm^{-1}	30.148	29.596	45.713
F(000)	4144.0	2064.0	5296.0
2Θ range /°	7.346 to 133.998	8.38 to 133.998	7.328 to 133.996
Reflections collected / unique	34230 / 12283	34014	35931
Data / restraints / parameters	12283 / 2094 / 749	12507 / 25 / 680	12483 / 0 / 694
R_1/wR_2 (I>2σ(I))^a	R_1 = 0.0368, wR_2 = 0.0886	R_1 = 0.0518, wR_2 = 0.1385	R_1 = 0.0597, wR_2 = 0.1603
R_1/wR_2 (all data)	R_1 = 0.0472, wR_2 = 0.0921	R_1 = 0.0568, wR_2 = 0.1423	R_1 = 0.0665, wR_2 = 0.1658
GooF (all data)^b	1.016	1.034	1.017
Max. peak/hole /e$^-$·Å^{-3}	1.95 / -1.29	3.49 / -1.57	4.43 / -2.77

^{a} R_1= = $\sum||F_o|-|F_c||/\sum|F_o|$; $wR_2 = \{\sum w[(F_o)^2-(F_c)^2]^2/\sum w[(F_o)^2]^2\}^{1/2}$

^{b} GooF = $\{\sum w[(Fo)^2-(Fc)^2]^2/(n-p)\}^{1/2}$

Table S2. Selected bond lengths (in Å) of the experiment (in **1-DMF**) and optimized geometries of $^{11}B_{3g}$-$[Fe_4@Sn_{18}]^{4-}$ at the M06-L/TZ2P level of theory.

	Experimental 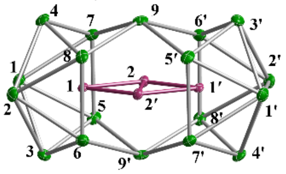	$^{11}B_{3g}$-$[Fe_4@Sn_{18}]^{4-}$ 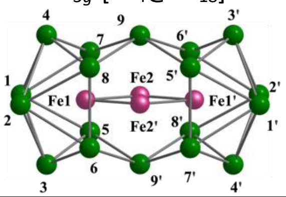
Sn1–Sn2	3.0436(7)	3.09
Sn1–Sn3	3.0544(7)	3.08
Sn1–Sn4	3.0355(7)	
Sn2–Sn3	3.0411(7)	
Sn2–Sn4	3.0562(7)	
Sn1–Sn5	3.1176(7)	3.17
Sn1–Sn7	3.1420(7)	
Sn2–Sn6	3.1361(7)	
Sn2–Sn8	3.1207(7)	
Sn3–Sn5	3.0441(7)	3.12
Sn3–Sn6	3.0487(7)	
Sn4–Sn7	3.0460(7)	
Sn4–Sn8	3.0722(7)	
Sn5–Sn7	3.0184(7)	3.09
Sn6–Sn8	3.0245(7)	
Sn5–Sn9'	3.2511(7)	3.24
Sn6–Sn9'	3.2157(7)	
Sn7–Sn9	3.2179(7)	
Sn8–Sn9	3.2291(7)	
Fe1–Fe2	2.4315(14)	2.41
Fe1–Fe2'	2.4309(14)	
Fe2–Fe2'	2.500(2)	2.52
Fe1–Sn1	2.8637(11)	2.87
Fe1–Sn2	2.8535(11)	
Fe1–Sn3	2.8345(11)	2.88
Fe1–Sn4	2.8349(11)	
Fe1–Sn5	2.8612(11)	2.93
Fe1–Sn6	2.8618(11)	
Fe1–Sn7	2.8644(11)	
Fe1–Sn8	2.8674(11)	
Fe2'–Sn5	2.7182(11)	2.72
Fe2'–Sn7	2.7150(11)	
Fe2–Sn8	2.7017(12)	
Fe2–Sn9	2.7176(12)	2.75
Fe2'–Sn9	2.7522(12)	

Table S3. Selected bond lengths (in Å) of the experiment and optimized geometries of $^{11}B_{1g}$-$[Fe_4@Pb_{18}]^{4-}$ at the M06-L/TZ2P level of theory.

	Experimental 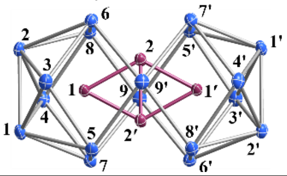	$^{11}B_{1g}$-$[Fe_4@Pb_{18}]^{4-}$ 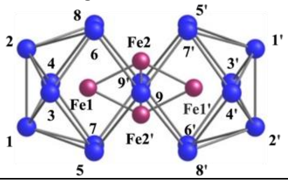
Pb1–Pb2	3.1784(7)	3.22
Pb1–Pb3	3.1394(7)	3.24
Pb1–Pb4	3.1653(7)	
Pb2–Pb3	3.1418(6)	
Pb2–Pb4	3.1527(7)	
Pb1–Pb5	3.2322(6)	3.35
Pb1–Pb7	3.2706(7)	
Pb2–Pb6	3.2848(6)	
Pb2–Pb8	3.2472(7)	
Pb3–Pb5	3.1892(7)	3.24
Pb3–Pb6	3.1551(7)	
Pb4–Pb7	3.1669(7)	
Pb4–Pb8	3.1771(7)	
Pb5–Pb7	3.2091(7)	3.28
Pb6–Pb8	3.2232(7)	
Pb5–Pb9'	3.2866(7)	3.32
Pb6–Pb9'	3.2747(7)	
Pb7–Pb9	3.2864(7)	
Pb8–Pb9	3.2580(7)	
Fe1–Fe2	2.431(2)	2.44
Fe1–Fe2'	2.437(2)	
Fe2–Fe2'	2.328(4)	2.29
Fe1–Pb1	2.9241(18)	2.97
Fe1–Pb2	2.9311(17)	
Fe1–Pb3	2.9734(19)	3.03
Fe1–Pb4	2.9742(18)	
Fe1–Pb5	2.9413(17)	3.02
Fe1–Pb6	2.9736(19)	
Fe1–Pb7	2.9578(19)	
Fe1–Pb8	2.9308(17)	
Fe2–Pb5	2.7944(19)	2.85
Fe2–Pb6	2.7906(19)	
Fe2–Pb7	2.8313(19)	
Fe2'–Pb8	2.8147(18)	
Fe2–Pb9	2.8729(19)	2.90
Fe2'–Pb9	2.8922(19)	

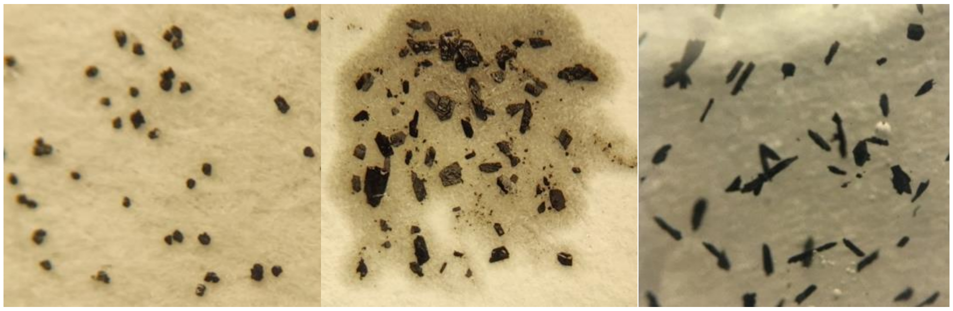

Figure S1. Crystal image of compounds **1** (left), **1-DMF** (middle) and **2** (right).

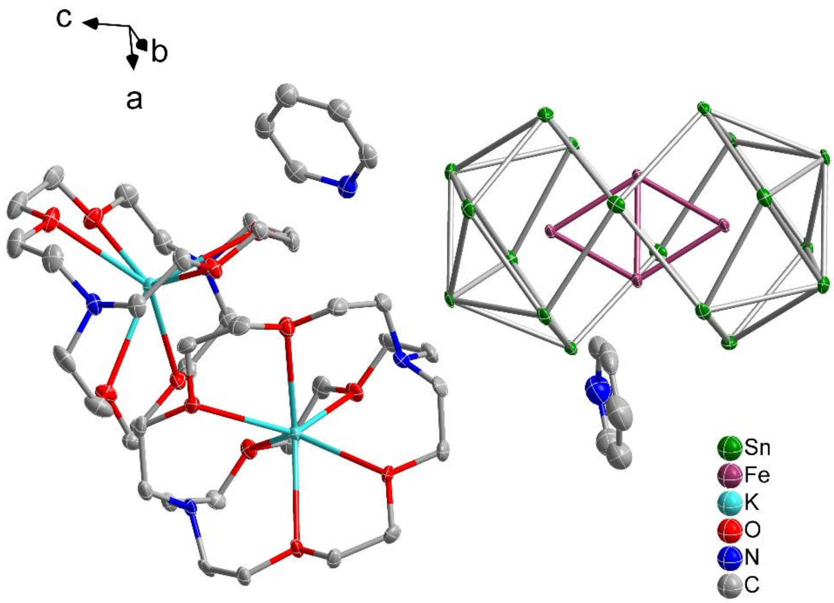

Figure S2. Asymmetric unit of **1**. The disorder positions (atoms and bonds) given in semi-transparent mode (60%).

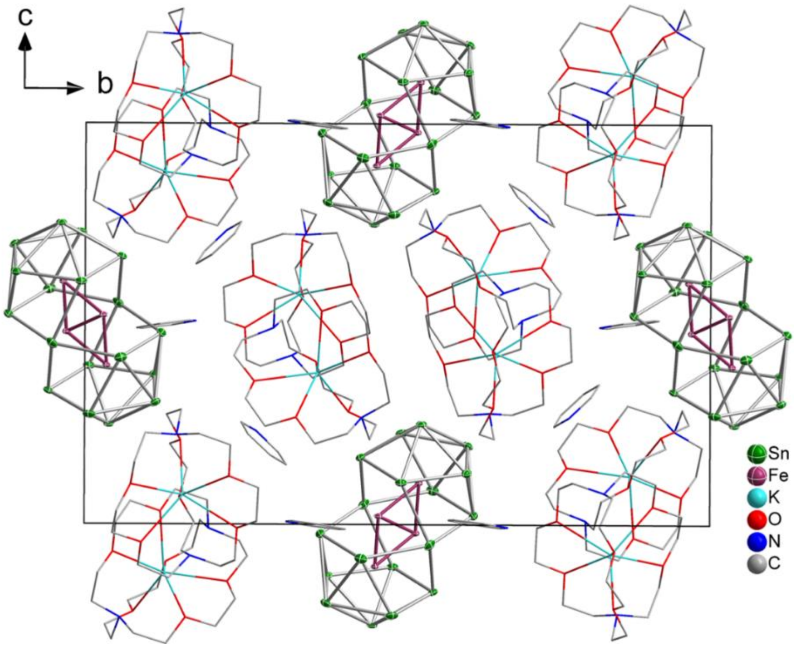

Figure S3. View of the unit cell of **1**. The disorder of Sn atoms is omitted here for clarity.

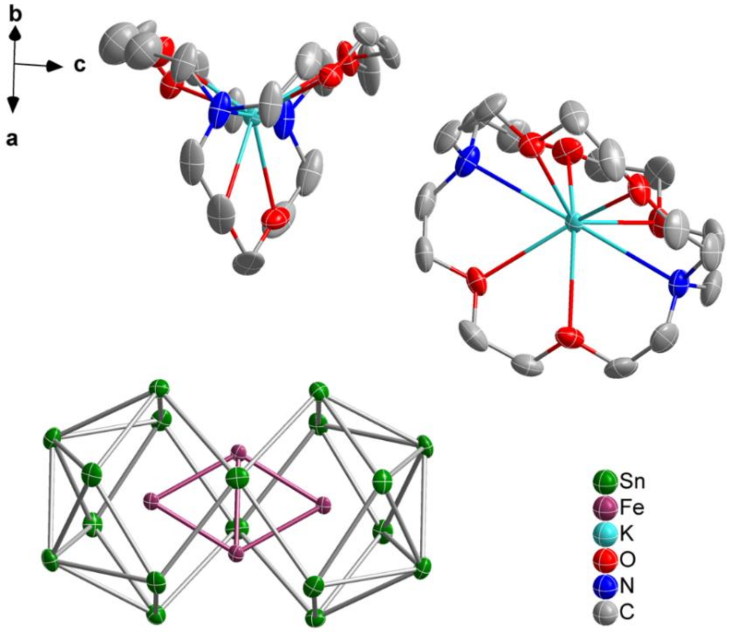

Figure S4. Asymmetric unit of **1-DMF**. The minor components (H atoms) in the cluster site are omitted for clarity.

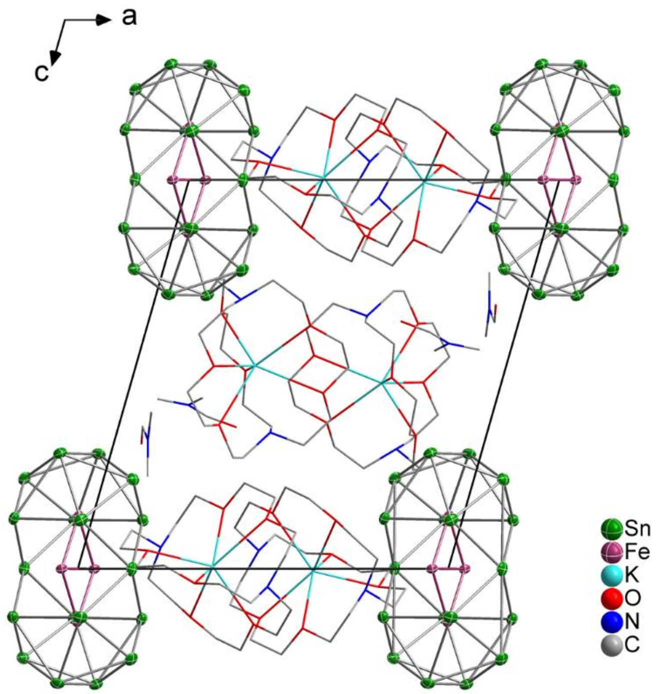

Figure S5. View of the unit cell of **1-DMF**. Minor components (H atoms) in the cluster site are omitted for clarity.

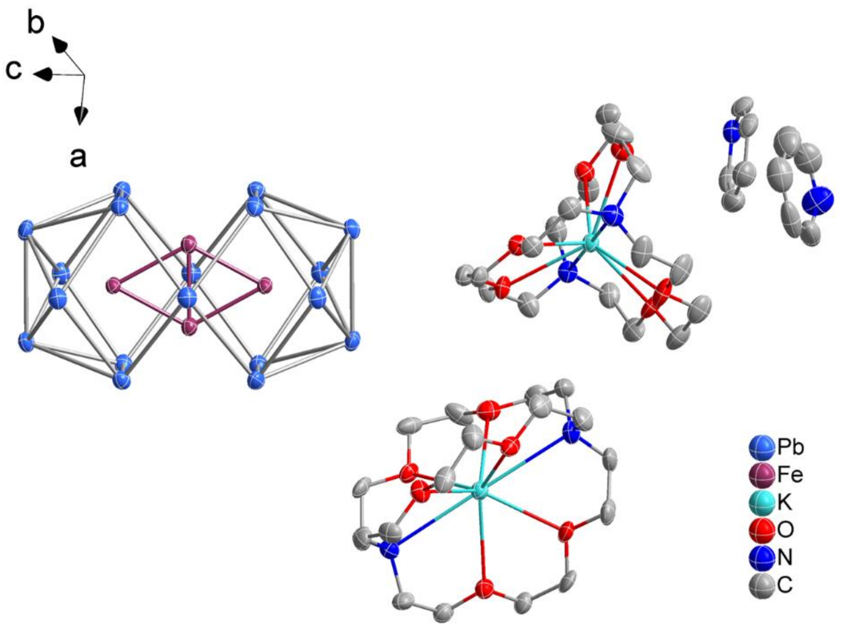

Figure S6. Asymmetric unit of **2** with the cluster fragment. Thermal ellipsoids are drawn at 50% probability. The minor components are omitted for clarity.

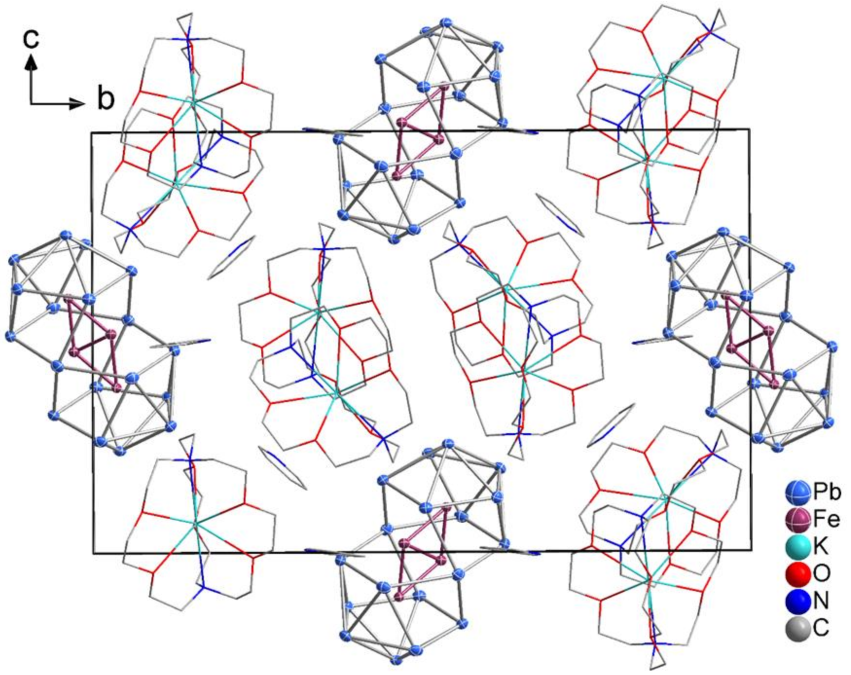

Figure S7. View of the unit cell of **2**. Minor components (H atoms) in the cluster site are omitted for clarity.

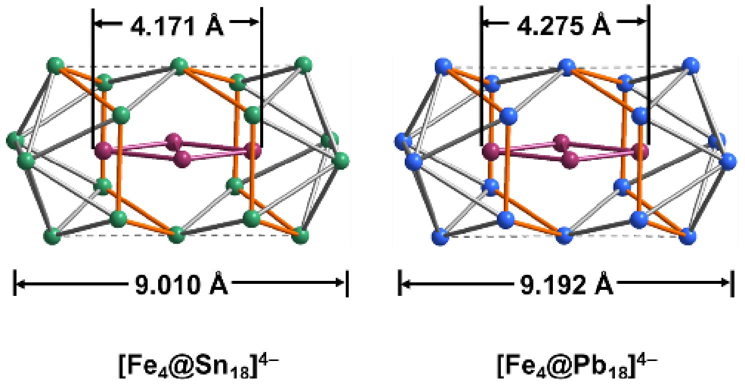

Figure S8. Axial distances of Fe motifs in compounds **1**-**2**, and the dimensions of anions $[Fe_4@Sn_{18}]^{4-}$, and $[Fe_4@Pb_{18}]^{4-}$.

3. ESI-MS Studies

The ESI-MS of the DMF solution of the crystals of **1** (Figure S9) and **2** (Figure S13) were performed under the condition of rapid injection in an inert atmosphere, indicating that $[Fe_4@Sn_{18}]^{2-}$ (m/z = 1179.9856), $[Fe_4@Sn_{18}]^{-}$ (m/z = 2359.9674), $[K(2.2.2\text{-crtpt})Fe_4@Sn_{18}]^{-}$ (m/z = 2775.1872), $[Fe_4@Pb_{18}]^{2-}$ (m/z = 1976.6402) and $[Fe@Pb_{12}]^{-}$ (m/z = 2542.6259) are stable in solution. Measured and simulated isotope distributions for all two species are shown in the Figures S10-12, S14, and S15.

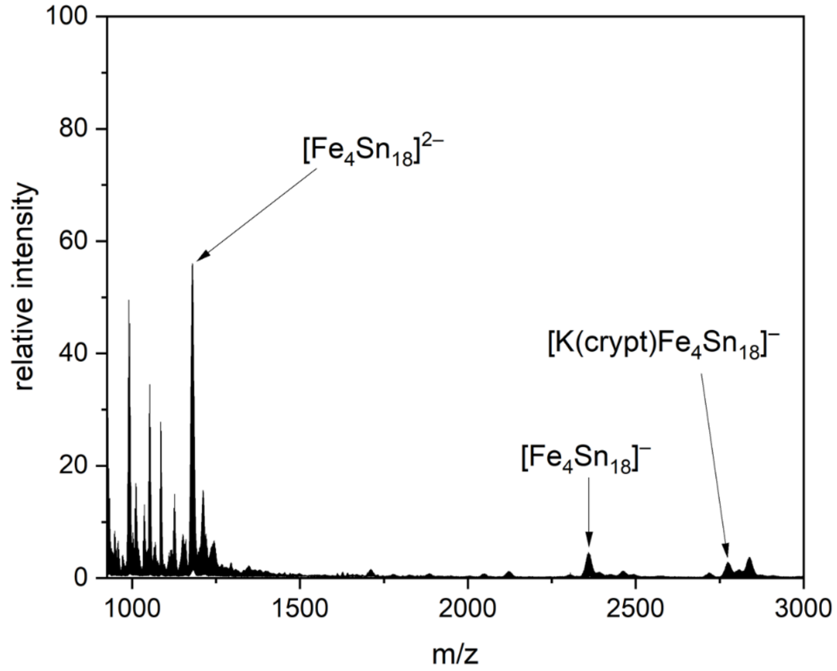

Figure S9. Overview ESI mass spectrum in negative ion mode recorded immediately upon injection of a fresh DMF solution of **1**.

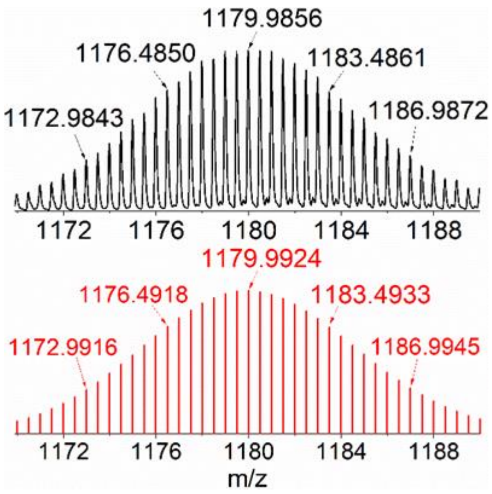

Figure S10. Measured (top) and simulated (bottom) spectrum of the fragment $[Fe_4Sn_{18}]^{2-}$.

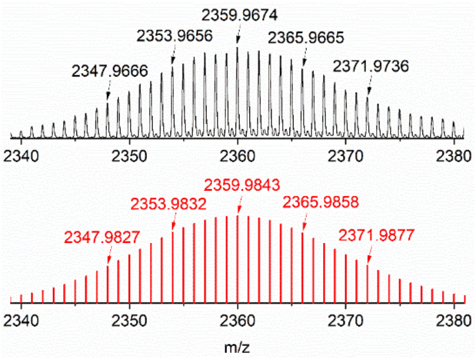

Figure S11. Measured (top) and simulated (bottom) spectrum of the fragment $[Fe_4Sn_{18}]^-$.

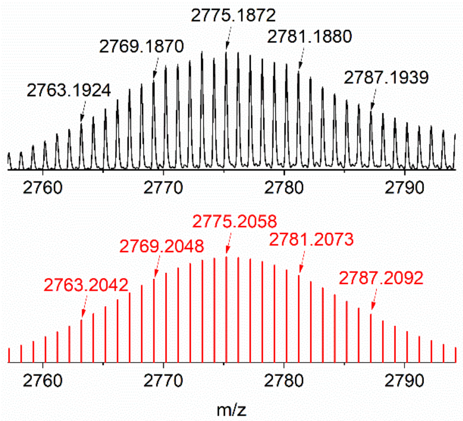

Figure S12. Measured (top) and simulated (bottom) spectrum of the fragment [K(2.2.2-crtpt)$Fe_4Sn_{18}]^-$.

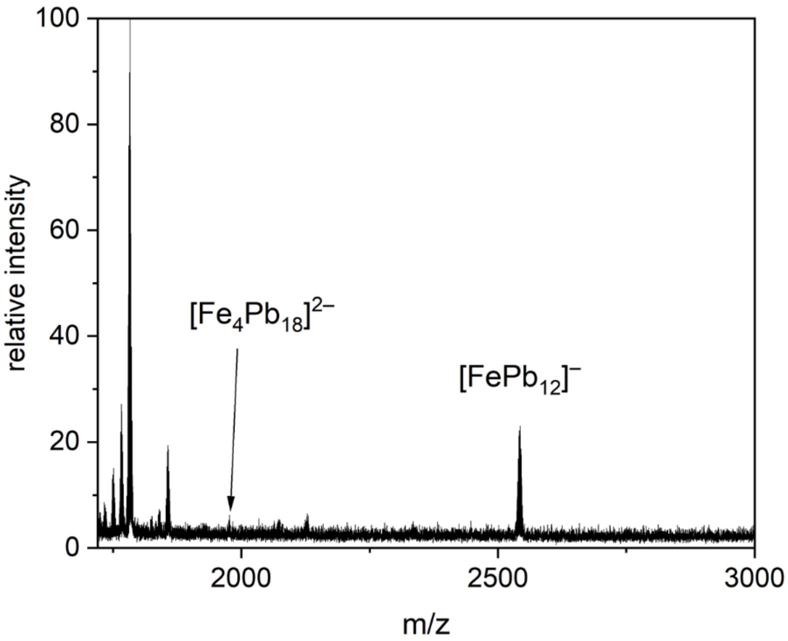

Figure S13. Overview ESI mass spectrum in negative ion mode recorded immediately upon injection of a fresh DMF solution of **2**.

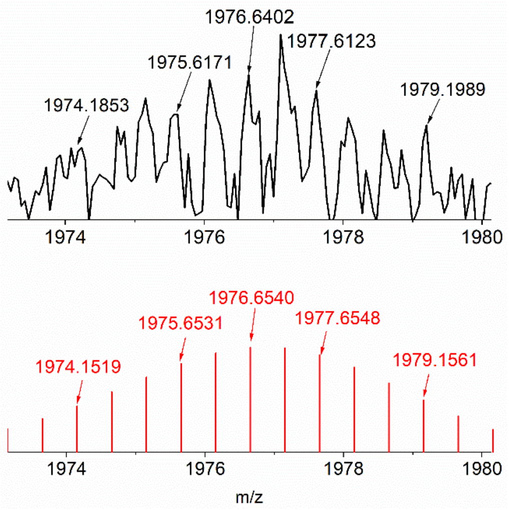

Figure S14. Measured (top) and simulated (bottom) spectrum of the fragment $[Fe_4Pb_{18}]^{2-}$.

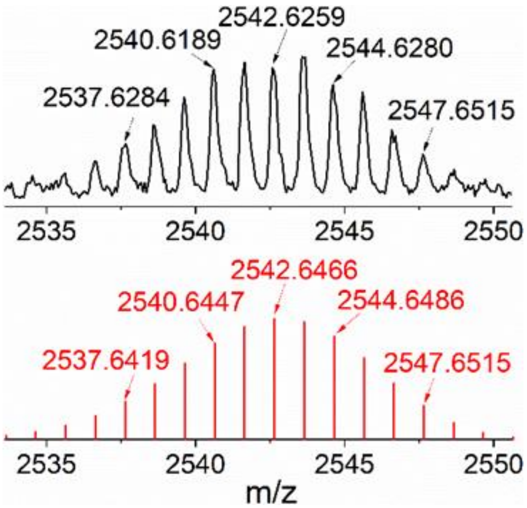

Figure S15. Measured (top) and simulated (bottom) spectrum of the fragment $[FePb_{12}]^{-}$.

4. Energy Dispersive X-ray (EDX) Spectroscopic Analysis

EDX analysis on **1** and **2** (Figure S16-S18) was performed by a scanning electron microscope (FE-SEM, JEOL JSM-7800F, Japan). Data acquisition was performed with an acceleration voltage of 15 kV and an accumulation time of 60 s. For **1-DMF** and **2**, a deviation in quantity K was observed in the EDX representation, which may be due to the irregular surface of a crystals after exposure to air.

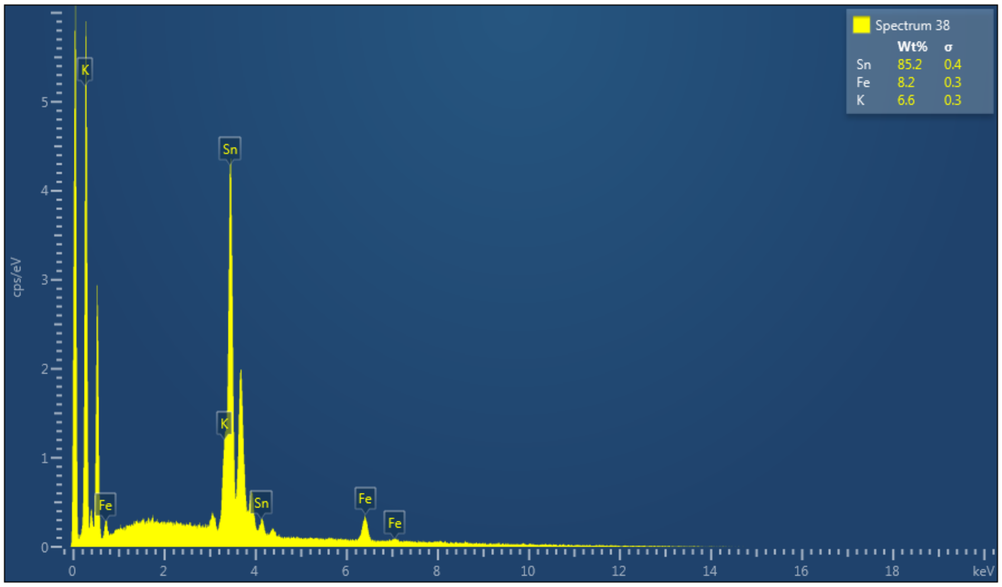

Figure S16. EDX analysis of **1**.

Element	wt%	σ	Observed / Calculated Element Ratio
K	6.6	0.3	4.3/4.0
Fe	8.2	0.3	3.7/4.0
Sn	85.2	0.4	18.3/18.0

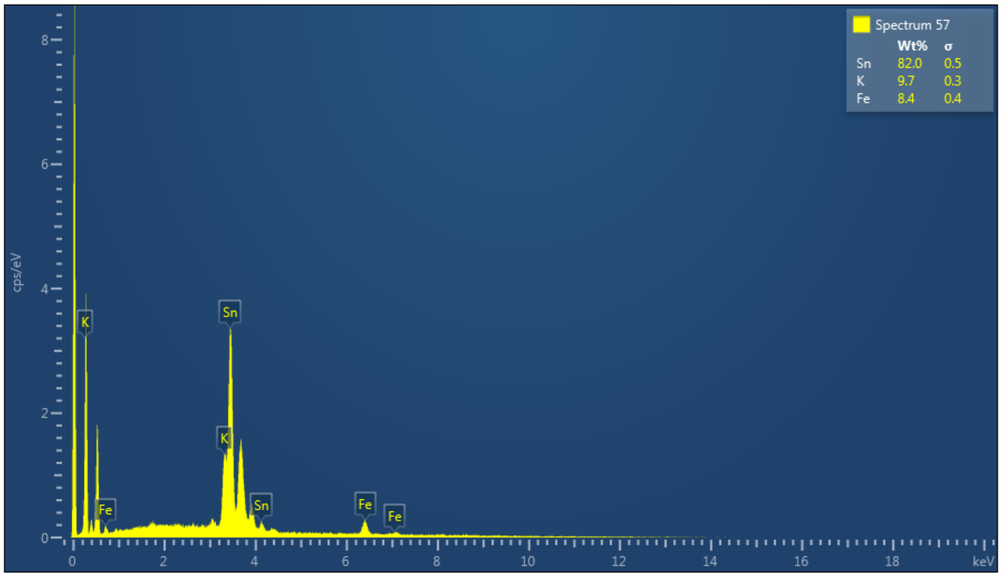

Figure S17. EDX analysis of **1-DMF**.

Element	wt%	σ	Observed / Calculated Element Ratio
K	9.7	0.3	6.5/4.0
Fe	8.4	0.4	3.9/4.0
Sn	82.0	0.5	18.0/18.0

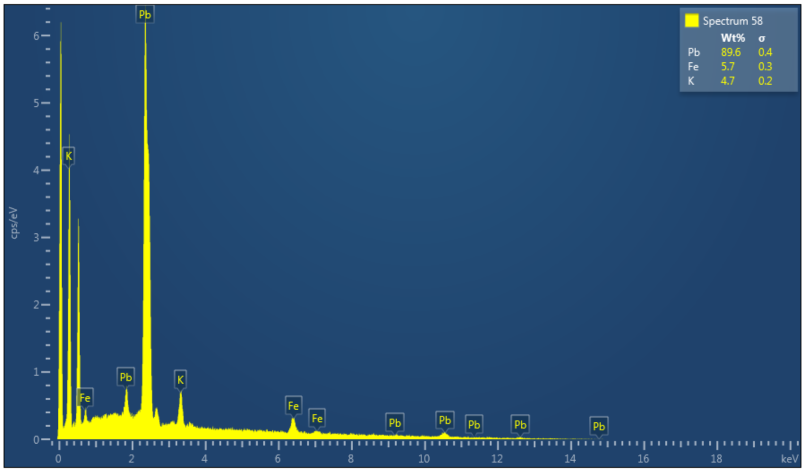

Figure S18. EDX analysis of **2**.

Element	wt%	σ	Observed / Calculated Element Ratio
K	4.7	0.2	5.0/4.0
Fe	5.7	0.3	4.2/4.0
Pb	89.6	0.4	18.0/18.0

5. Magnetometry using the Superconducting Quantum Interference Device (SQUID)

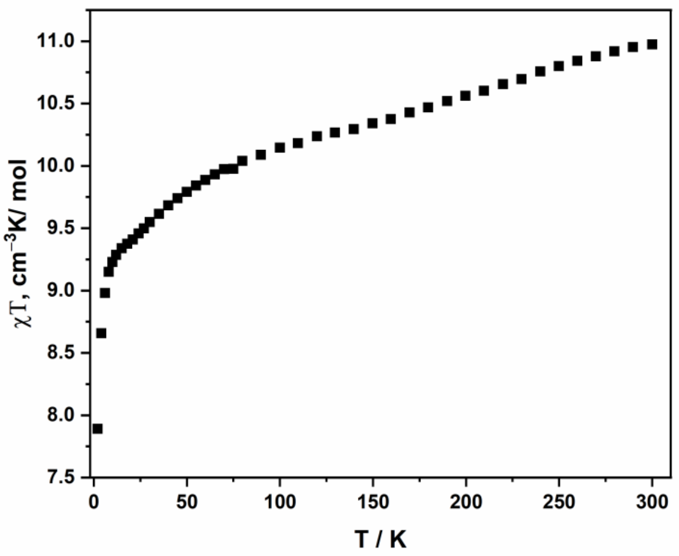

Figure S19. Temperature-dependent SQUID magnetometry data for **1** measured over the temperature range of 1.8 to 300 K.

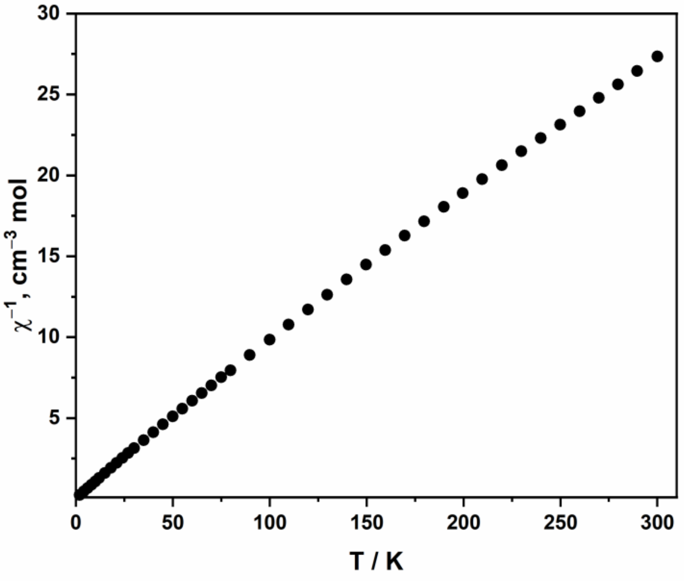

Figure S20. Temperature dependent, inverse solid state magnetic susceptibility for **1** collected under an applied field of H = 1000 Oe.

6. Computational Details

Functional Dependence of Spin State Energies

In order to explore the dependence of the computed energies and geometries on functional choice, we have repeated the calculations reported in the main text (done with the M06-L functional) with a generalized gradient approximation (GGA) functional, PBE, and also its hybrid version, PBE0. The energies are summarized in Table S4 and S5. Note that we were unable to converge on the *S* = 4 or *S* = 6 states for the PBE0 functional. The PBE and M06-L functional give a broadly consistent picture of the relative energies of the two states with *S* = 5, $^{11}B_{3g}$ and $^{11}B_{1g}$. For $[Fe_4Sn_{18}]^{4-}$, the $^{11}B_{1g}$ state is the most stable, but the gap to the $^{11}B_{3g}$ alternative is even smaller for PBE (0.02 eV) than for M06-L (0.05 eV). This trend goes further with the PBE0 functional, where the $^{11}B_{3g}$ state is now more stable than $^{11}B_{1g}$ by 0.07 eV. The states with *S* = 4 and *S* = 6 are considerably less stable than those with *S* = 5 for both M06-L and PBE, but the latter tends to stabilize the *S* = 4 state and destabilize *S* = 6, relative to the two states with *S* = 5. Similar patterns are apparent for $[Fe_4Pb_{18}]^{4-}$, in Table S5, where the PBE functional stabilizes $^{11}B_{3g}$ relative to $^{11}B_{1g}$, although the latter is clearly more stable. PBE also stabilizes *S* = 4 and destabilizes *S* = 6 relative to the two *S* = 5 states, but the *S* = 5 remains clearly more stable. Thus, whilst there is considerable fluctuation in the relative energies, the conclusion that the *S* = 5 state is the best candidate for the ground state of both Fe_4 clusters appears robust.

Table S4. Bond energies of different spin states of $[Fe_4Sn_{18}]^{4-}$ with M06-L, PBE,[5] PBE0[6-7] functionals. All energies are in eV.

	S=4	S=5 ($^{11}B_{3g}$)	S=5 ($^{11}B_{1g}$)	S=6
M06-L	-121.6021 ($^{9}A_{g}$)	-122.1392	-122.1927	-121.9378 ($^{13}B_{2g}$)
PBE	-112.5093 ($^{9}B_{1u}$)	-112.6542	-112.6735	-112.2424 ($^{13}B_{3g}$)
PBE0		- 151.1726	- 151.0985	

Table S5. Bond energies of different spin states of $[Fe_4Pb_{18}]^{4-}$ with M06-L and PBE functionals. All energies are in eV.

	S=4 ($^{9}B_{2u}$)	S=5 ($^{11}B_{3g}$)	S=5 ($^{11}B_{1g}$)	S=6 ($^{13}B_{3g}$)
M06-L	-116.0026	-116.6095	-116.7923	-116.6074
PBE	-106.1882	-106.3245	-106.4770	-106.0472

Discussion of the charge state of the E_{18} cage

In our recent paper discussing the structure and properties of $[Fe_3Sn_{18}]^{4-}$,[8] we argued that the presence of partially fused Sn_9 polyhedra was indicative of a Sn_{18}^{6-} charge state. This contrasts with the situation in $[Cd(NiSn_9)_2]^{6-}$,[9] where the two polyhedral are completely separated with a charge of 8- and also with the completely fused limit found in $[Pd_2Sn_{18}]^{4-}$,[10] where the charge is 4-. Lin and co-workers have also explored the fusion of Sn_9 units using their principal interacting orbitals (PIO) approach.[11,12,13] The question that we address here is which charge state is appropriate to the E_{18} units in the $[Fe_4Sn_{18}]^{4-}$ and $[Fe_4Pb_{18}]^{4-}$ clusters discussed in this paper. The

stability of the Cu analogues, $[Cu_4Sn_{18}]^{4-}$ and $[Cu_4Pb_{18}]^{4-}$, where the Cu ions are unambiguously in the +1 charge state (as judged by the filled 3d band in the projected density of states plots) provides strong circumstantial evidence for the presence of an $[E_{18}]^{8-}$ unit to balance the charge.[14] We can reinforce this argument by extracting the Sn_{18} units from converged geometries for $[Fe_4Sn_{18}]^{4-}$, $[Cd(NiSn_9)_2]^{6-}$ and $[Pd_2Sn_{18}]^{4-}$ and performing single point calculations on each structure in the 4- and 8- charge states (see Table S6). At the 4- charge level, it is clear that the fused geometry of the $[Pd_2Sn_{18}]^{4-}$ cluster is optimal. At the 8- charge state, the geometries obtained from $[Fe_4Sn_{18}]^{4-}$ and $[Cd(NiSn_9)_2]^{6-}$ are both more stable than that from $[Pd_2Sn_{18}]^{4-}$. The different number of metal ions in $[Fe_4Sn_{18}]^{4-}$ and $[Cd(NiSn_9)_2]^{6-}$ makes it difficult to make a direct energetic comparison, but it is clear that the geometry of the Sn_{18} cluster in the former is in some sense adapted to an 80-electron count ($[Sn_{18}]^{8-}$).

Table S6. Bond energies of charged Sn_{18} from the optimized endohedral Sn_{18}-based clusters. All energies are in eV.

Charge State	Cage from $[Pd_2Sn_{18}]^{4-}$/eV	Cage from $[Cd(NiSn_9)_2]^{6-}$/eV	Cage from $[Fe_4Sn_{18}]^{4-}$/eV
4–	-77.24	-76.39	-75.26
8–	-82.71	-87.29	-84.11

To develop this idea further, we have extended the analysis in our recent paper on $[Fe_3Sn_{18}]^{4-}$ using the Walsh diagram approach applied in our recent work on $[Fe_3Sn_{18}]^{4-}$, where we showed that the coalescence of the E_9 polyhedra across the series $[Cd(NiSn_9)_2]^{6-}$, $[Fe_3Sn_{18}]^{4-}$, $[Pd_2Sn_{18}]^{4-}$ causes the destabilisation of two orbitals, and hence a reduction in characteristic charge from 8- to 4- (Figure S21). It is not immediately obvious how to link the structure with two separated Sn_9 units to the fused D_{2h}-symmetric geometry found in $[Fe_4Sn_{18}]^{4-}$, but this can be achieved using the D_{3d}-symmetric $[Fe_3Sn_{18}]^{4-}$ as an intermediate, as shown in the Walsh diagram below. The first step, the partial fusion of two Sn_9 units, results in the destabilisation of one orbital and hence a reduction in characteristic charge from 8- to 6-. The second step, converting the structure found in $[Fe_3Sn_{18}]^{4-}$ to that found in $[Fe_4Sn_{18}]^{4-}$ involves a rotation of the atoms in the central Sn_6 unit, and results in the net stabilisation of two orbitals ($7b_{1u}$ and $5b_{2g}$) and the destabilisation of one ($9a_g$), restoring the characteristic 8- charge. In this light, we see that the separated E_9 limit and the D_{2h}-symmetric isomers of E_{18} are both adapted to accommodate an electron count of precisely 80, and hence a net 8- charge, albeit in different ways: the balance between the two forms will then be determined by the availability of the metal cations.

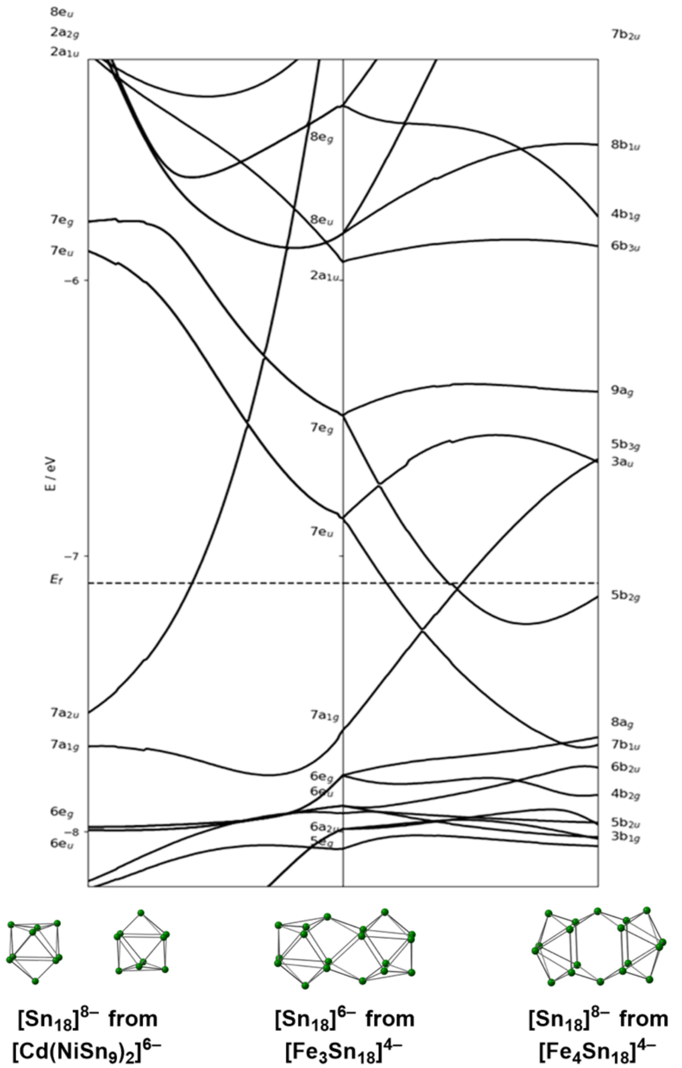

Figure S21. The Walsh diagram connecting three Sn_{18} clusters - the separated $[Sn_{18}]^{8-}$ from optimized $[Cd(NiSn_9)_2]^{6-}$, partially fused $[Sn_{18}]^{6-}$ from optimized $[Fe_3Sn_{18}]^{4-}$ and fused $[Sn_{18}]^{8-}$ from optimized $[Fe_4Sn_{18}]^{4-}$. The orbitals vary with the geometry changes, possessing 40, 38 and 40 low-lying orbitals for three Sn_{18} cages respectively.

Electronic structure analysis using DOS, PDOS and OPDOS

The description of the bonding presented in the main text is based on a careful analysis of the Kohn-Sham orbitals and also the densities of states (DOS), projected densities of states (PDOS) and overlap-projected densities of states (OPDOS) shown in Figure S22. The eigenvalue spectrum of these molecular clusters is, of course, discrete, so a brief comment on why we adopt an analysis based on levels broadened into a density of states using an arbitrary Lorentzian line width is in order. The PDOS defines the contribution of different atomic levels to the DOS at a given energy while the OPDOS, sometimes referred to at the Crystal Orbital Overlap Population (COOP) in a solid-state context, defines the degree of bonding (positive) or antibonding (negative) character for a given atom pair as a function of energy. The trio of DOS, PDOS and OPDOS provide

a graphical picture of (a) the energies of the orbitals, (b) their composition (PDOS) and (c) their bonding/antibonding character (OPDOS). It therefore allows for a rapid assessment of, for example, the degree of Fe-Fe bonding character in a given configuration. The PDOS in Figure S23 is projected onto Fe/Cu 3d and Sn 5p/Pb 6p states while the OPDOS is projected onto the M1-M2 and M2-M2′ pairs (only the spin-β manifolds are shown for the latter). As discussed in our previous paper, the PDOS of the Cu cluster is highly characteristic of closed-shell Cu^{+}: a narrow occupied band of Cu 3d levels between −3 and −4 eV (relative to the Fermi level) contains all Cu-Cu linear combinations from bonding (positive OPDOS) to antibonding (negative OPDOS). The band of Sn 5p or Pb 6p levels lies above Cu 3d, between −3 eV and the Fermi level, and there is limited mixing of Cu and tetrel levels: the interaction between Cu and Sn can be considered, to a good first approximation, as an ionic one. The assignment of an oxidation state to Fe in $[Fe_4Sn_{18}]^{4-}$ and $[Fe_4Pb_{18}]^{4-}$ is less straightforward, but the striking similarities between the Sn 5p PDOS of $[Cu_4Sn_{18}]^{4-}$ and $[Fe_4Sn_{18}]^{4-}$ is a first indication that the E_{18} units share a common oxidation level and so, therefore, must the transition metal ions (*i.e.* Cu_4^{4+} and Fe_4^{4+}). The OPDOS shows that the doubly-occupied levels are largely Fe1-Fe2 (blue OPDOS curve) and Fe2-Fe2′ (red OPDOS curve) bonding while the singly-occupied and vacant levels are largely antibonding, and the excess of bonding over antibonding electrons gives rise to net Fe-Fe bonding. We can identify three distinct bonding/antibonding orbital pairs that make a substantial net contributions to the Fe2-Fe2′ bonding in both the $^{11}B_{1g}$ and $^{11}B_{3g}$ states: the $8a_g^2 8b_{2u}^1$ configuration (Fe2-Fe2′ σ), the $4b_{1u}^2 5b_{3g}^1$ configuration (out-of-plane π) and the $7b_{3u}^2 7b_{1g}^0$ configuration (in-plane π). The dominant peaks in the Fe1-Fe2 OPDOS, in contrast, come from the $5b_{2g}^2 4a_u^1$ configuration (out-of-plane π). The differences between the $^{11}B_{3g}$ and $^{11}B_{1g}$ states then lie in the reversal of the $6b_{1g}$ and $4b_{3g}$ orbitals, the first of which has significant Fe2-Fe2′ in-plane π^* character (negative OPDOS): the strengthening of this in-plane component in the $^{11}B_{1g}$ state is responsible for the contraction of the Fe2-Fe2′ bond in $[Fe_4Pb_{18}]^{4-}$. The differences between Cu and Fe, and between Sn and Pb, are captured in Table 2, where the bond lengths, Mayer bond orders (MBO), and delocalization indices (DI), both of which correlate with bond strength, are collected. The values for the Cu clusters again establish a baseline corresponding to weak interactions of a cuprophilic type. The Mayer bond orders and delocalization indices are systematically larger for the Fe-Fe bonds compared to their Cu-Cu counterparts, with the most striking differences coming in the $^{11}B_{1g}$ states, where the Fe2-Fe2′ bonds have bond orders greater than 0.8 compared to values of ∼0.6 in the $^{11}B_{3g}$ state. The contraction of the Fe2-Fe2′ bond in $[Fe_4Pb_{18}]^{4-}$ compared to $[Fe_4Sn_{18}]^{4-}$ appears, therefore, to mark a very substantial increase in π component of the bond.

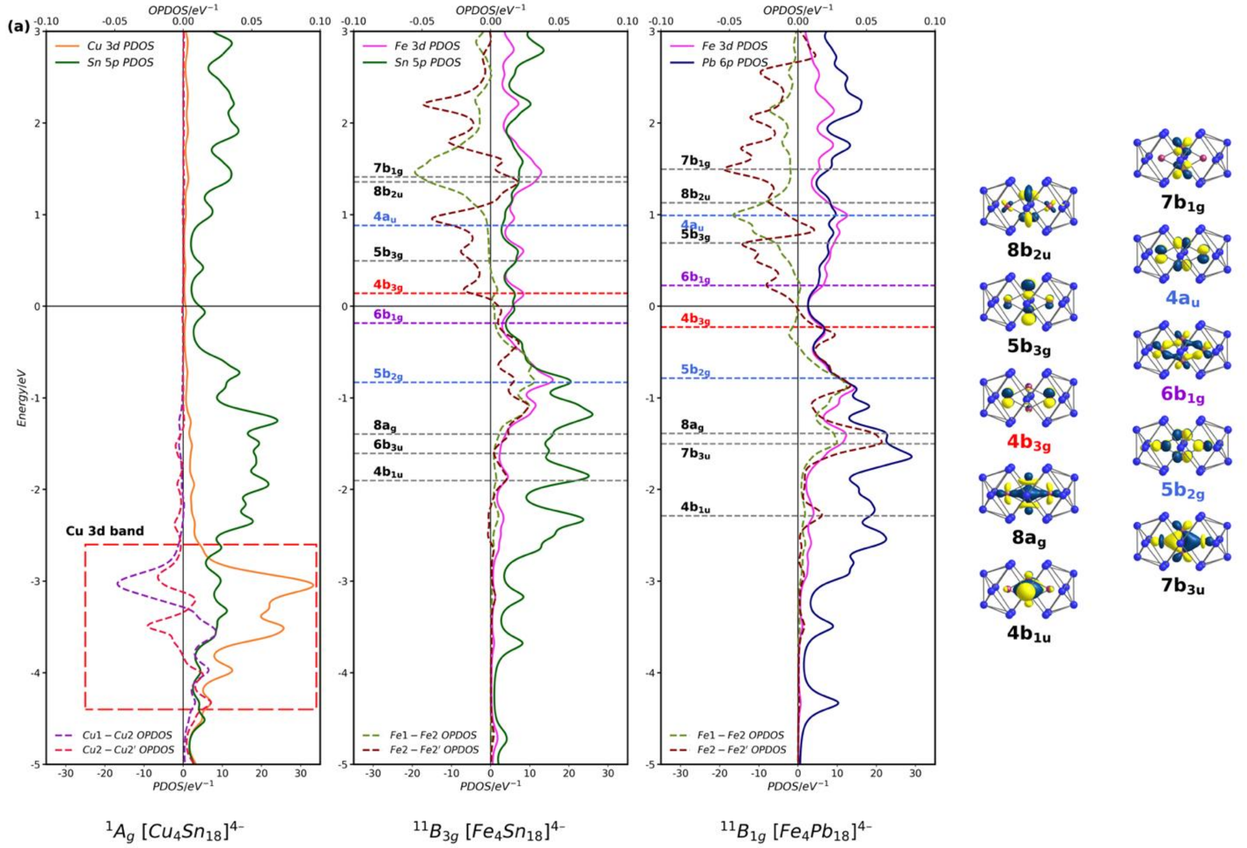

Figure S22. The PDOS and OPDOS for $[Cu_4Sn_{18}]^{4-}$, and for the spin-b manifolds of $[Fe_4Sn_{18}]^{4-}$ and $[Fe_4Pb_{18}]^{4-}$. The two spin components, α and β, of the PDOS and OPDOS for $[Fe_4Sn_{18}]^{4-}$ and $[Fe_4Pb_{18}]^{4-}$ are collected in Figures S22 and S23 (only the spin-β components are shown in Figure S22). In both cases, the Fe-Fe antibonding orbitals lie largely above the Fermi level in the spin-β set, but below it in spin-α. This leads to an accumulation of bonding character.

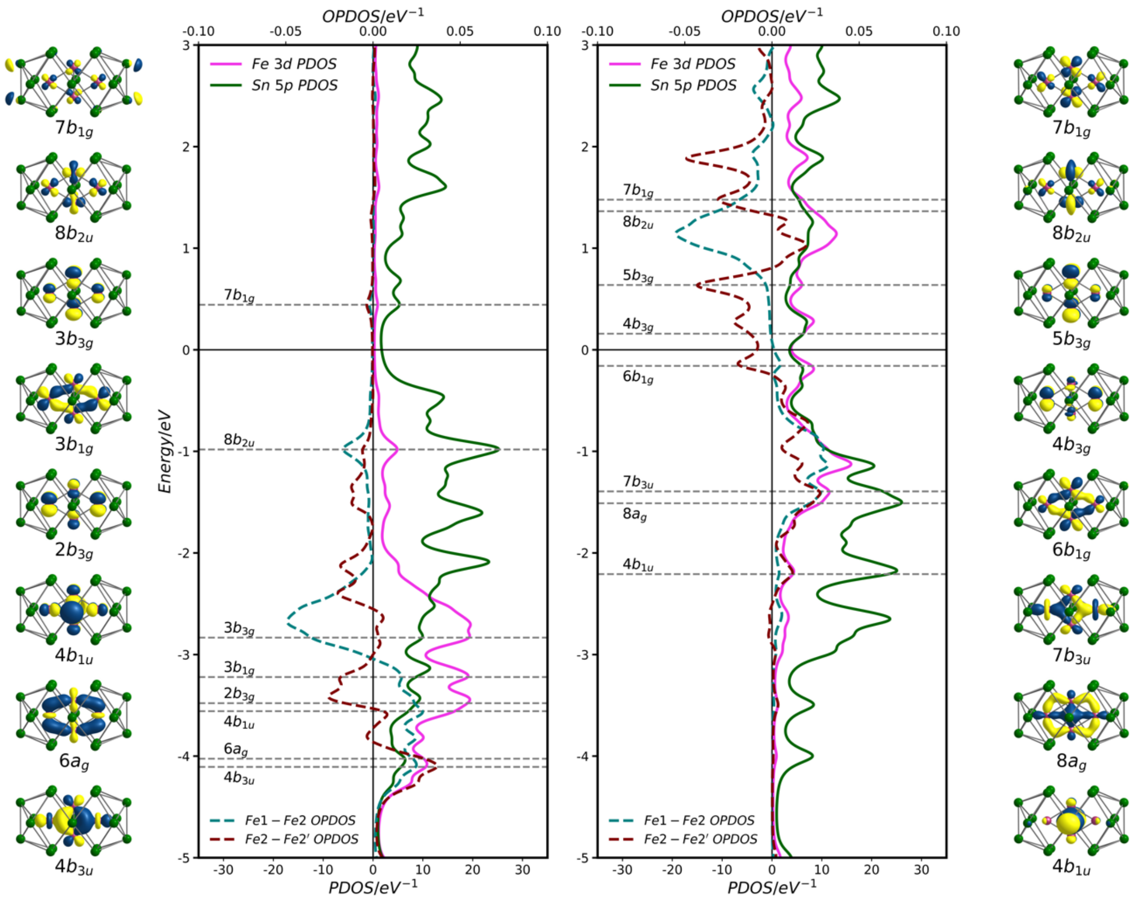

Figure S23. The PDOS and OPDOS for both majority and minority spin of $[Fe_4Sn_{18}]^{4-}$. The energy levels that mediate the σ and π contributions to Fe2-Fe2’ bonds are labelled, as are the two orbitals that switch occupancies between the $^{11}B_{1g}$ and $^{11}B_{3g}$ states.

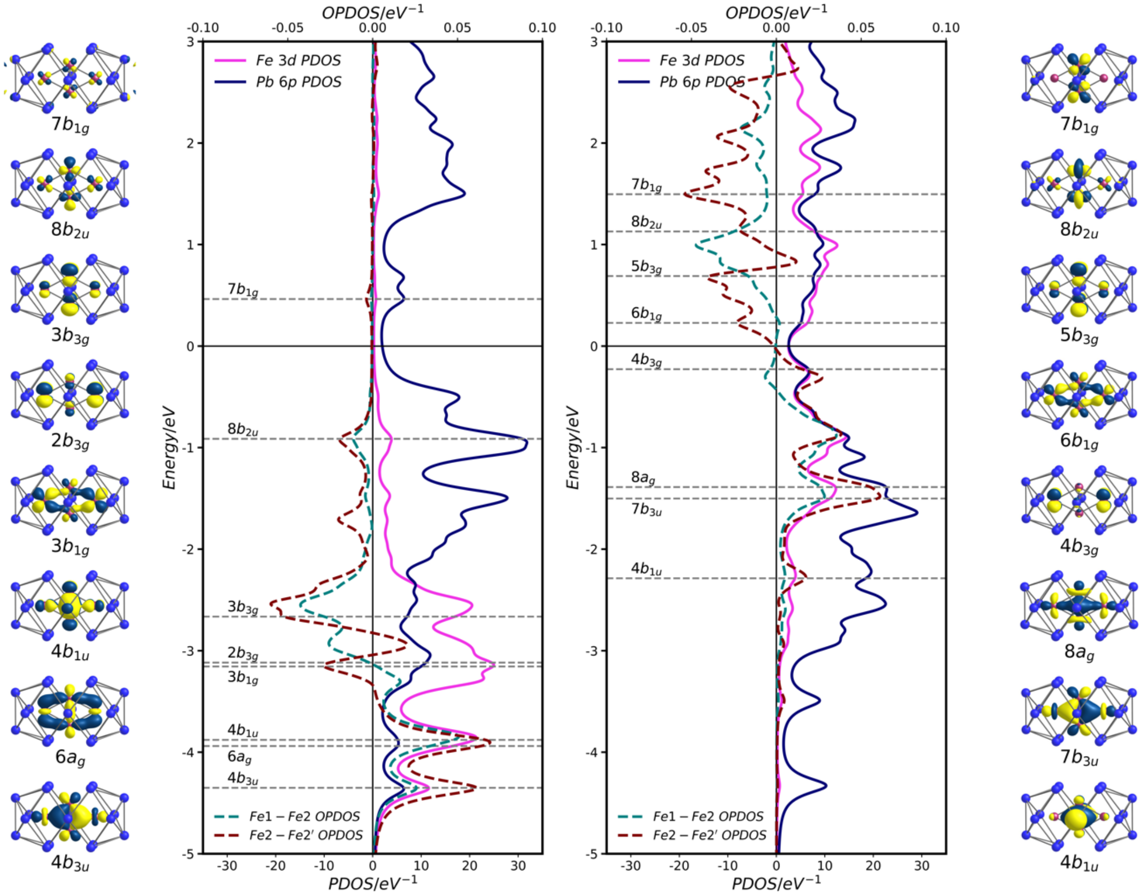

Figure S24. The PDOS and OPDOS for both majority and minority spin of $[Fe_4Pb_{18}]^{4-}$. The energy levels that mediate the σ and π contributions to Fe2-Fe2’ bonds are labelled, as are the two orbitals that switch occupancies between the $^{11}B_{1g}$ and $^{11}B_{3g}$ states.

Electron Localization Functions (ELF) and density distribution analysis

The emergence of Fe-Fe bonding is also illustrated graphically by the electron density distributions in Figure S25 and the electron localization functions (ELF), shown in Figure S26. The accumulation of density at the mid-point of the Fe1-Fe2 and Fe2-Fe2’ vectors in both $[Fe_4Sn_{18}]^{4-}$ and $[Fe_4Pb_{18}]^{4-}$ is clear, as is the positive (orange and red) ELF basin along the Fe1-Fe2 vectors and in the center of Fe_4 rhombus.

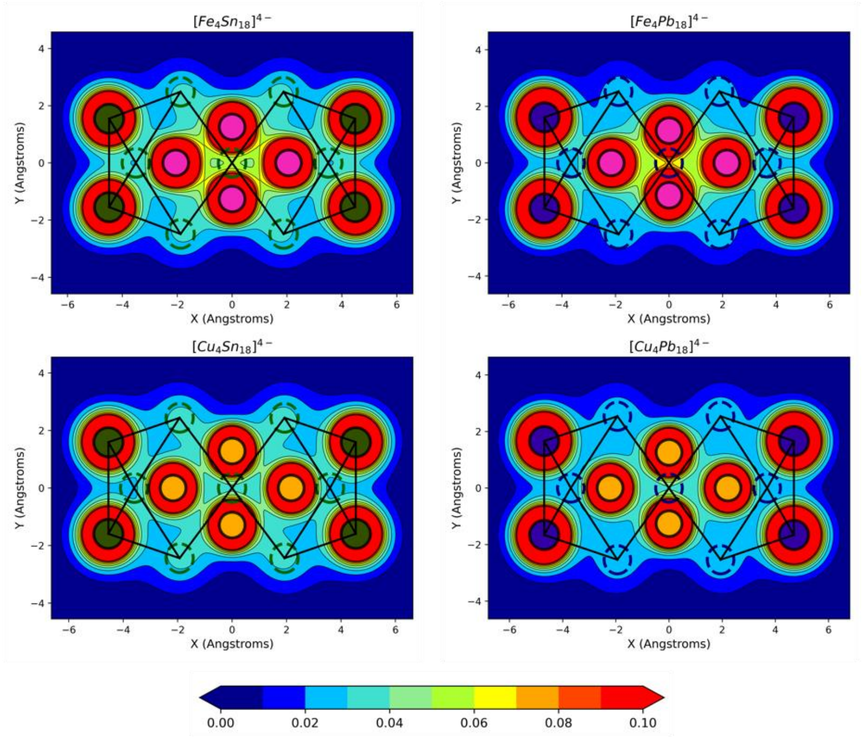

Figure S25. Electron density distributions of $[Fe_4E_{18}]^{4-}$ and $[Cu_4E_{18}]^{4-}$ (E = Sn, Pb) on the M_4 planes. The density increases with the color from blue to red. The contour range is 0 to 0.1.

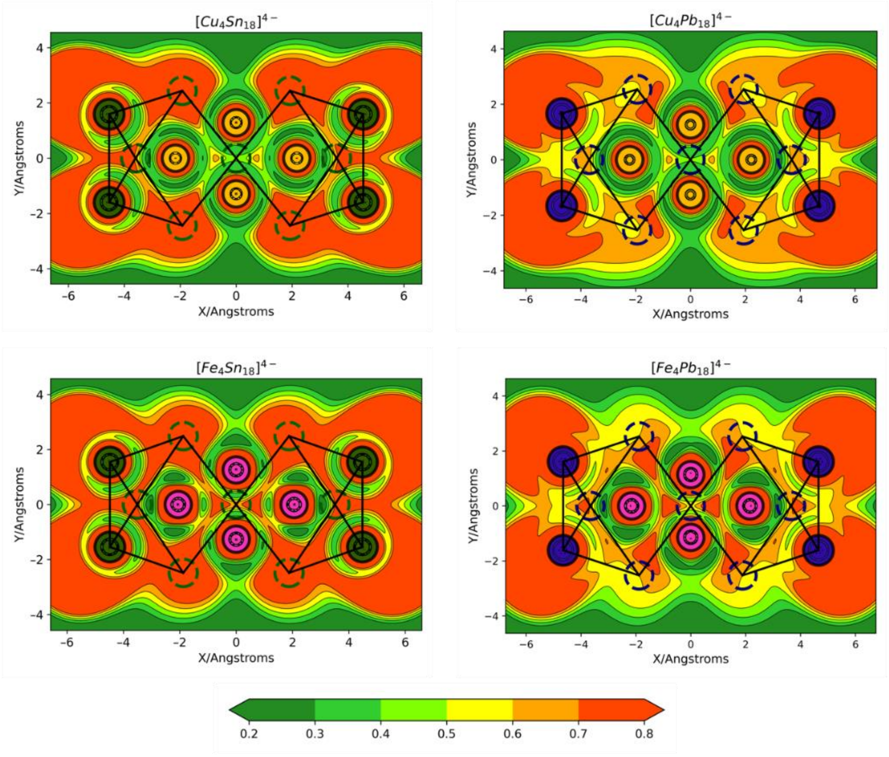

Figure S26. Electron localization functions (ELF) for $[Cu_4E_{18}]^{4-}$ and $[Fe_4E_{18}]^{4-}$ and on the M_4 planes. The contour range is from 0.2 to 0.8.

Table S7. Mayer bond orders (MBO), delocalisation indices (DI) and ellipticities (ε) of the Fe-Fe bond critical points.

			M-M	MBO	DI	ε
$[Fe_4Sn_{18}]^{4-}$	$^{11}B_{1g}$	Fe1-Fe2	2.42	0.82	0.68	0.91
		Fe2-Fe2'	2.31	0.82	0.97	0.45
	$^{11}B_{3g}$	Fe1-Fe2	2.41	0.85	0.74	0.26
		Fe2-Fe2'	2.52	0.51	0.64	2.11
$[Fe_4Pb_{18}]^{4-}$	$^{11}B_{1g}$	Fe1-Fe2	2.44	0.69	0.70	1.02
		Fe2-Fe2'	2.28	0.91	1.00	0.46
	$^{11}B_{3g}$	Fe1-Fe2	2.43	0.74	0.75	0.31
		Fe2-Fe2'	2.48	0.62	0.70	1.77
$[Cu_4Sn_{18}]^{4-}$	$^{1}A_{g}$	Cu1-Cu2	2.52	0.41	0.32	0.20
		Cu2-Cu2'	2.58	0.10	0.29	0.15
$[Cu_4Pb_{18}]^{4-}$	$^{1}A_{g}$	Cu1-Cu2	2.55	0.31	0.32	0.23
		Cu2-Cu2'	2.52	0.18	0.32	0.22

Coordinates of Computational Results (Appendix)

The optimized cartesian coordinates of the corresponding stationary points of $^{11}B_{1g}$ and $^{11}B_{3g}$ states of $[Fe_4Sn_{18}]^{4-}$ and $[Fe_4Pb_{18}]^{4-}$, with bond energies provided.

***D*$_{2h}$-$[Fe_4Sn_{18}]^{4-}$ ($^{11}B_{1g}$) M06-L**; S = 5; Bond Energy = -122.1927 eV

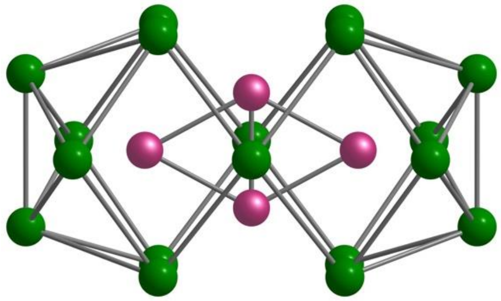

Fe	-2.130715	-0.000000	0.000000
Fe	-0.000000	1.153535	0.000000
Fe	0.000000	-1.153535	0.000000
Fe	2.130715	0.000000	0.000000
Sn	-4.507484	-1.532943	0.000000
Sn	-4.507484	1.532943	0.000000
Sn	-3.561771	0.000000	-2.521662
Sn	-3.561771	0.000000	2.521662
Sn	-1.861710	-2.409333	-1.579990
Sn	-1.861710	-2.409333	1.579990
Sn	-1.861710	2.409333	-1.579990
Sn	-1.861710	2.409333	1.579990
Sn	0.000000	-0.000000	-2.536886
Sn	0.000000	0.000000	2.536886
Sn	1.861710	-2.409333	-1.579990
Sn	1.861710	-2.409333	1.579990
Sn	1.861710	2.409333	-1.579990
Sn	1.861710	2.409333	1.579990
Sn	3.561771	-0.000000	-2.521662
Sn	3.561771	-0.000000	2.521662
Sn	4.507484	-1.532943	0.000000
Sn	4.507484	1.532943	0.000000

***D*$_{2h}$-$[Fe_4Sn_{18}]^{4-}$ ($^{11}B_{3g}$) M06-L**; S = 5; Bond Energy = -122.1392 eV

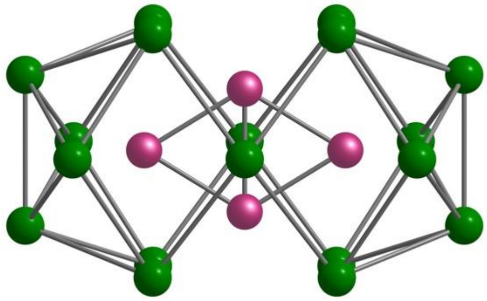

Fe	-2.054520	-0.000000	0.000000
Fe	-0.000000	1.259551	0.000000
Fe	0.000000	-1.259551	0.000000
Fe	2.054520	0.000000	0.000000
Sn	-4.481842	-1.545122	0.000000
Sn	-4.481842	1.545122	0.000000
Sn	-3.506483	0.000000	-2.482056
Sn	-3.506483	0.000000	2.482056
Sn	-1.877336	-2.486922	-1.545396
Sn	-1.877336	-2.486922	1.545396
Sn	-1.877336	2.486922	-1.545396
Sn	-1.877336	2.486922	1.545396
Sn	0.000000	-0.000000	2.443174
Sn	0.000000	0.000000	-2.443174
Sn	1.877336	-2.486922	-1.545396
Sn	1.877336	-2.486922	1.545396
Sn	1.877336	2.486922	-1.545396
Sn	1.877336	2.486922	1.545396
Sn	3.506483	-0.000000	-2.482056
Sn	3.506483	-0.000000	2.482056
Sn	4.481842	-1.545122	0.000000
Sn	4.481842	1.545122	0.000000

***D_{2h}*-$[Fe_4Pb_{18}]^{4-}$ ($^{11}B_{1g}$) M06-L**; S = 5; Bond Energy = -116.7924 eV

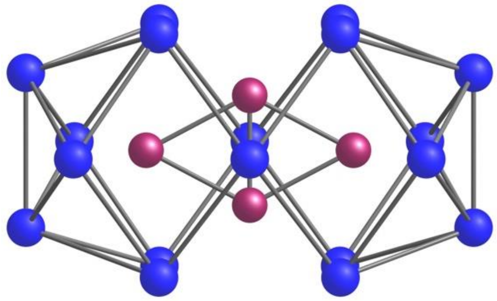

Fe	-2.155771	0.000000	0.000000
Fe	0.000000	-1.142377	0.000000
Fe	0.000000	1.142377	0.000000
Fe	2.155771	0.000000	0.000000

Pb	-4.653745	-1.611333	0.000000
Pb	-4.653745	1.611333	0.000000
Pb	-3.656158	0.000000	-2.630213
Pb	-3.656158	0.000000	2.630213
Pb	-1.882569	-2.527431	-1.637573
Pb	-1.882569	-2.527431	1.637573
Pb	-1.882569	2.527431	-1.637573
Pb	-1.882569	2.527431	1.637573
Pb	0.000000	0.000000	-2.667369
Pb	0.000000	0.000000	2.667369
Pb	1.882569	-2.527431	-1.637573
Pb	1.882569	-2.527431	1.637573
Pb	1.882569	2.527431	-1.637573
Pb	1.882569	2.527431	1.637573
Pb	3.656158	0.000000	-2.630213
Pb	3.656158	0.000000	2.630213
Pb	4.653745	-1.611333	0.000000
Pb	4.653745	1.611333	0.000000

***D_{2h}*-$[Fe_4Pb_{18}]^{4-}$ ($^{11}B_{3g}$) M06-L**; S = 5; Bond Energy = -116.6095 eV

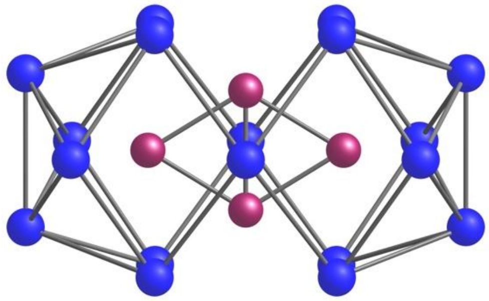

Fe	-2.086286	0.000000	0.000000
Fe	0.000000	-1.237961	0.000000
Fe	0.000000	1.237961	0.000000
Fe	2.086286	0.000000	0.000000
Pb	-4.628587	-1.626312	0.000000
Pb	-4.628587	1.626312	0.000000
Pb	-3.601909	0.000000	-2.591589
Pb	-3.601909	0.000000	2.591589
Pb	-1.895771	-2.603710	-1.613053
Pb	-1.895771	-2.603710	1.613053
Pb	-1.895771	2.603710	-1.613053
Pb	-1.895771	2.603710	1.613053
Pb	0.000000	0.000000	-2.582918
Pb	0.000000	0.000000	2.582918
Pb	1.895771	-2.603710	-1.613053
Pb	1.895771	-2.603710	1.613053

Pb	1.895771	2.603710	-1.613053
Pb	1.895771	2.603710	1.613053
Pb	3.601909	0.000000	-2.591589
Pb	3.601909	0.000000	2.591589
Pb	4.628587	-1.626312	0.000000
Pb	4.628587	1.626312	0.000000

The optimized cartesian coordinates of 9et and 13et of $[Fe_4Sn_{18}]^{4-}$ and $[Fe_4Pb_{18}]^{4-}$, with bond energies provided. Stationary points were confirmed.

D_{2h}-$[Fe_4Sn_{18}]^{4-}$ (^9A_g) M06-L; S = 4; Bond Energy = -121.6021 eV

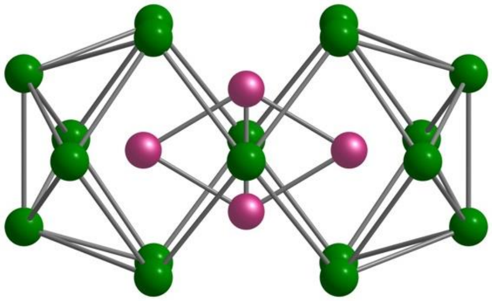

Fe	-2.070819	-0.000000	0.000000
Fe	-0.000000	-1.236232	0.000000
Fe	0.000000	1.236232	0.000000
Fe	2.070819	0.000000	0.000000
Sn	-4.474137	-1.547014	0.000000
Sn	-4.474137	1.547014	0.000000
Sn	-3.540801	0.000000	-2.503259
Sn	-3.540801	0.000000	2.503259
Sn	-1.866031	-2.416192	-1.581613
Sn	-1.866031	-2.416192	1.581613
Sn	-1.866031	2.416192	-1.581613
Sn	-1.866031	2.416192	1.581613
Sn	0.000000	-0.000000	-2.457478
Sn	0.000000	0.000000	2.457478
Sn	1.866031	-2.416192	-1.581613
Sn	1.866031	-2.416192	1.581613
Sn	1.866031	2.416192	-1.581613
Sn	1.866031	2.416192	1.581613
Sn	3.540801	-0.000000	-2.503259
Sn	3.540801	-0.000000	2.503259
Sn	4.474137	-1.547014	0.000000
Sn	4.474137	1.547014	0.000000

D_{2h}-$[Fe_4Sn_{18}]^{4-}$ ($^{13}B_{2g}$) M06-L; S = 6; Bond Energy = -121.9378 eV

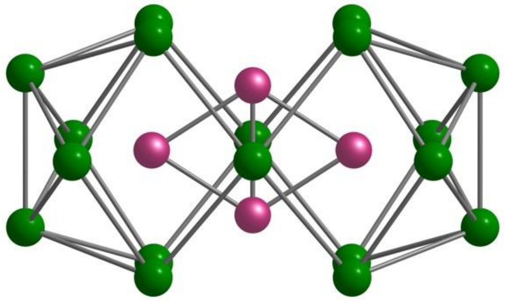

Fe	-2.042279	-0.000000	0.000000
Fe	-0.000000	1.314034	0.000000
Fe	0.000000	-1.314034	0.000000
Fe	2.042279	0.000000	0.000000
Sn	-4.595467	-1.562767	0.000000
Sn	-4.595467	1.562767	0.000000
Sn	-3.637559	-0.000000	-2.477535
Sn	-3.637559	-0.000000	2.477535
Sn	-2.004794	-2.431050	-1.543943
Sn	-2.004794	-2.431050	1.543943
Sn	-2.004794	2.431050	-1.543943
Sn	-2.004794	2.431050	1.543943
Sn	0.000000	-0.000000	2.420789
Sn	0.000000	0.000000	-2.420789
Sn	2.004794	-2.431050	-1.543943
Sn	2.004794	-2.431050	1.543943
Sn	2.004794	2.431050	-1.543943
Sn	2.004794	2.431050	1.543943
Sn	3.637559	0.000000	-2.477535
Sn	3.637559	0.000000	2.477535
Sn	4.595467	-1.562767	0.000000
Sn	4.595467	1.562767	0.000000

D_{2h}-$[Fe_4Pb_{18}]^{4-}$ ($^9B_{1u}$) M06-L; S = 4; Bond Energy = -116.0026 eV

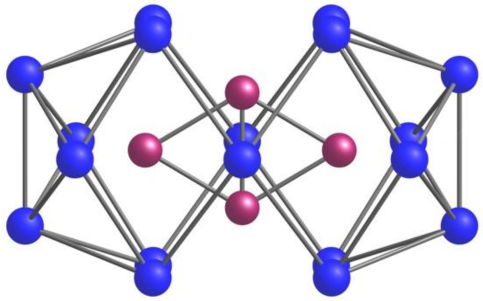

Fe	-2.069987	-0.000000	0.000000
Fe	0.000000	-1.230955	0.000000
Fe	0.000000	1.230955	-0.000000
Fe	2.069987	0.000000	0.000000

Pb	-4.632883	-1.616400	0.000000
Pb	-4.632883	1.616400	-0.000000
Pb	-3.555866	-0.000000	2.597529
Pb	-3.555866	0.000000	-2.597529
Pb	-1.903795	-2.594250	-1.614491
Pb	-1.903795	-2.594250	1.614491
Pb	-1.903795	2.594250	-1.614491
Pb	-1.903795	2.594250	1.614491
Pb	0.000000	-0.000000	2.628148
Pb	0.000000	0.000000	-2.628148
Pb	1.903795	-2.594250	-1.614491
Pb	1.903795	-2.594250	1.614491
Pb	1.903795	2.594250	-1.614491
Pb	1.903795	2.594250	1.614491
Pb	3.555866	-0.000000	2.597529
Pb	3.555866	0.000000	-2.597529
Pb	4.632883	-1.616400	0.000000
Pb	4.632883	1.616400	-0.000000

***D_{2h}*-[Fe_4Pb_{18}]$^{4-}$ ($^{13}B_{3g}$) M06-L**; S = 6; Bond Energy = -116.6074 eV

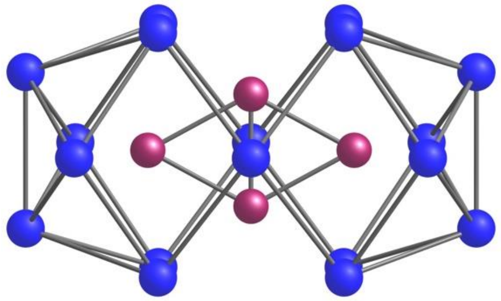

Fe	-2.161117	0.000000	0.000000
Fe	0.000000	-1.175322	0.000000
Fe	0.000000	1.175322	-0.000000
Fe	2.161117	-0.000000	0.000000
Pb	-4.719939	-1.640677	0.000000
Pb	-4.719939	1.640677	-0.000000
Pb	-3.690727	-0.000000	2.592842
Pb	-3.690727	0.000000	-2.592842
Pb	-1.944816	-2.560814	-1.612833
Pb	-1.944816	-2.560814	1.612833
Pb	-1.944816	2.560814	-1.612833
Pb	-1.944816	2.560814	1.612833
Pb	0.000000	-0.000000	2.602707
Pb	0.000000	0.000000	-2.602707
Pb	1.944816	-2.560814	-1.612833
Pb	1.944816	-2.560814	1.612833

Pb	1.944816	2.560814	-1.612833
Pb	1.944816	2.560814	1.612833
Pb	3.690727	-0.000000	2.592842
Pb	3.690727	0.000000	-2.592842
Pb	4.719939	-1.640677	0.000000
Pb	4.719939	1.640677	-0.000000

The optimized coordinates of $[Fe_4Sn_{18}]^{4-}$ and $[Fe_4Pb_{18}]^{4-}$ under other functionals (PBE and PBE0), with corresponding bond energies. Stationary points were confirmed.

***D_{2h}*-$[Fe_4Sn_{18}]^{4-}$ ($^{11}B_{1g}$) PBE**; S = 5; Bond Energy = -112.6735 eV

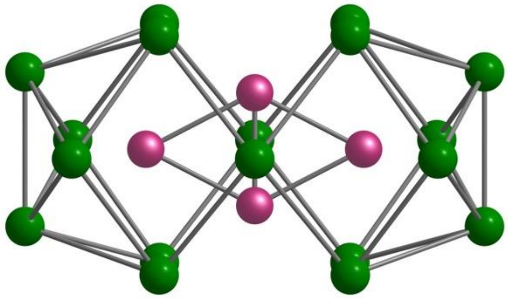

Fe	-2.154070	-0.000000	0.000000
Fe	0.000000	-1.118983	-0.000000
Fe	0.000000	1.118983	0.000000
Fe	2.154070	0.000000	0.000000
Sn	-4.543798	-1.531427	0.000000
Sn	-4.543798	1.531427	-0.000000
Sn	-3.610798	-0.000000	2.512863
Sn	-3.610798	0.000000	-2.512863
Sn	-1.887490	-2.369052	-1.567209
Sn	-1.887490	-2.369052	1.567209
Sn	-1.887490	2.369052	-1.567209
Sn	-1.887490	2.369052	1.567209
Sn	0.000000	-0.000000	-2.557180
Sn	0.000000	0.000000	2.557180
Sn	1.887490	-2.369052	-1.567209
Sn	1.887490	-2.369052	1.567209
Sn	1.887490	2.369052	-1.567209
Sn	1.887490	2.369052	1.567209
Sn	3.610798	-0.000000	2.512863
Sn	3.610798	0.000000	-2.512863
Sn	4.543798	-1.531427	0.000000
Sn	4.543798	1.531427	-0.000000

***D_{2h}*-$[Fe_4Sn_{18}]^{4-}$ ($^{11}B_{3g}$) PBE**; S = 5; Bond Energy = -112.6542 eV

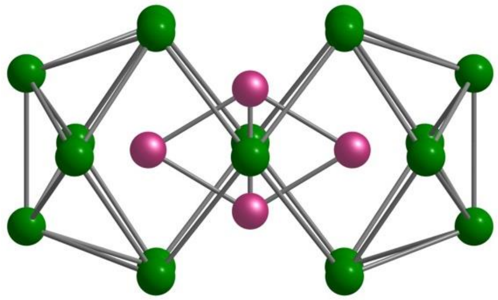

Fe	-2.056073	0.000000	0.000000
Fe	0.000000	-1.237769	0.000000
Fe	0.000000	1.237769	0.000000
Fe	2.056073	0.000000	0.000000
Sn	-4.508401	-1.546406	0.000000
Sn	-4.508401	1.546406	0.000000
Sn	-3.530526	0.000000	-2.472283
Sn	-3.530526	0.000000	2.472283
Sn	-1.896936	-2.457632	-1.530740
Sn	-1.896936	-2.457632	1.530740
Sn	-1.896936	2.457632	-1.530740
Sn	-1.896936	2.457632	1.530740
Sn	0.000000	0.000000	-2.455217
Sn	0.000000	0.000000	2.455217
Sn	1.896936	-2.457632	-1.530740
Sn	1.896936	-2.457632	1.530740
Sn	1.896936	2.457632	-1.530740
Sn	1.896936	2.457632	1.530740
Sn	3.530526	0.000000	-2.472283
Sn	3.530526	0.000000	2.472283
Sn	4.508401	-1.546406	0.000000
Sn	4.508401	1.546406	0.000000

***D_{2h}*-$[Fe_4Sn_{18}]^{4-}$ ($^9B_{2u}$) PBE**; S = 4; Bond Energy = -112.5093 eV

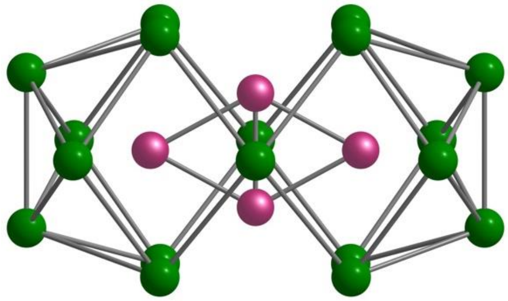

Fe	-2.076626	-0.000000	0.000000
Fe	0.000000	-1.129888	-0.000000
Fe	0.000000	1.129888	0.000000
Fe	2.076626	0.000000	0.000000

Sn	-4.519003	-1.538511	-0.000000
Sn	-4.519003	1.538511	0.000000
Sn	-3.590976	-0.000000	-2.505059
Sn	-3.590976	0.000000	2.505059
Sn	-1.884617	-2.376204	-1.543629
Sn	-1.884617	-2.376204	1.543629
Sn	-1.884617	2.376204	-1.543629
Sn	-1.884617	2.376204	1.543629
Sn	0.000000	-0.000000	-2.553677
Sn	0.000000	0.000000	2.553677
Sn	1.884617	-2.376204	-1.543629
Sn	1.884617	-2.376204	1.543629
Sn	1.884617	2.376204	-1.543629
Sn	1.884617	2.376204	1.543629
Sn	3.590976	-0.000000	-2.505059
Sn	3.590976	0.000000	2.505059
Sn	4.519003	-1.538511	-0.000000
Sn	4.519003	1.538511	0.000000

***D_{2h}*-[Fe_4Sn_{18}]$^{4-}$ ($^{13}B_{3g}$) PBE**; S = 6; Bond Energy = -112.2424 eV

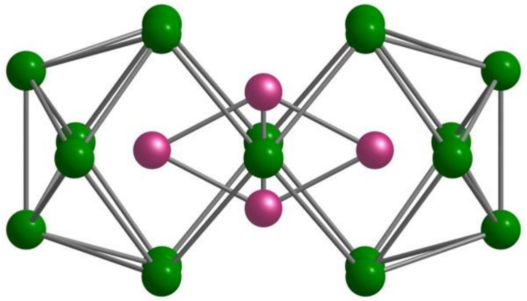

Fe	-2.181900	0.000000	0.000000
Fe	0.000000	-1.139100	0.000000
Fe	0.000000	1.139100	0.000000
Fe	2.181900	0.000000	0.000000
Sn	-4.646300	-1.556300	0.000000
Sn	-4.646300	1.556300	0.000000
Sn	-3.688000	0.000000	-2.482400
Sn	-3.688000	0.000000	2.482400
Sn	-1.992200	-2.373300	-1.536400
Sn	-1.992200	-2.373300	1.536400
Sn	-1.992200	2.373300	-1.536400
Sn	-1.992200	2.373300	1.536400
Sn	0.000000	0.000000	-2.488500
Sn	0.000000	0.000000	2.488500
Sn	1.992200	-2.373300	-1.536400
Sn	1.992200	-2.373300	1.536400

Sn	1.992200	2.373300	-1.536400
Sn	1.992200	2.373300	1.536400
Sn	3.688000	0.000000	-2.482400
Sn	3.688000	0.000000	2.482400
Sn	4.646300	-1.556300	0.000000
Sn	4.646300	1.556300	0.000000

***D_{2h}*-$[Fe_4Pb_{18}]^{4-}$ ($^{11}B_{1g}$) PBE**; S = 5; Bond Energy = -106.4770 eV

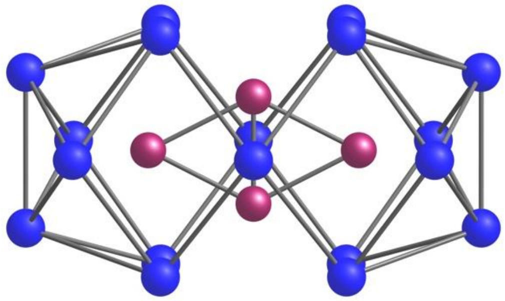

Fe	-2.165392	0.000000	0.000000
Fe	0.000000	-1.102186	0.000000
Fe	0.000000	1.102186	0.000000
Fe	2.165392	0.000000	0.000000
Pb	-4.684661	-1.602776	0.000000
Pb	-4.684661	1.602776	0.000000
Pb	-3.712302	0.000000	-2.617373
Pb	-3.712302	0.000000	2.617373
Pb	-1.910978	-2.475537	-1.622707
Pb	-1.910978	-2.475537	1.622707
Pb	-1.910978	2.475537	-1.622707
Pb	-1.910978	2.475537	1.622707
Pb	0.000000	0.000000	-2.699830
Pb	0.000000	0.000000	2.699830
Pb	1.910978	-2.475537	-1.622707
Pb	1.910978	-2.475537	1.622707
Pb	1.910978	2.475537	-1.622707
Pb	1.910978	2.475537	1.622707
Pb	3.712302	0.000000	-2.617373
Pb	3.712302	0.000000	2.617373
Pb	4.684661	-1.602776	0.000000
Pb	4.684661	1.602776	0.000000

***D_{2h}*-$[Fe_4Pb_{18}]^{4-}$ ($^{11}B_{3g}$) PBE**; S = 5; Bond Energy = - 106.3127 eV

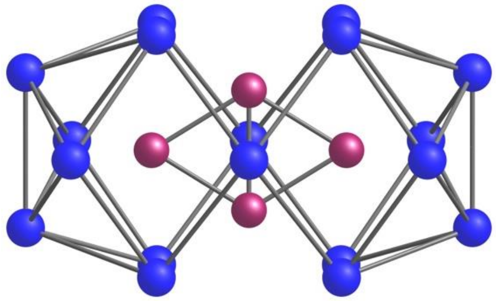

Fe	-2.068925	0.000000	0.000000
Fe	0.000000	-1.212783	-0.000000
Fe	0.000000	1.212783	0.000000
Fe	2.068925	-0.000000	0.000000
Pb	-4.642258	-1.617941	0.000000
Pb	-4.642258	1.617941	-0.000000
Pb	-3.621362	-0.000000	2.576613
Pb	-3.621362	0.000000	-2.576613
Pb	-1.910992	-2.570283	-1.597852
Pb	-1.910992	-2.570283	1.597852
Pb	-1.910992	2.570283	-1.597852
Pb	-1.910992	2.570283	1.597852
Pb	0.000000	-0.000000	2.598173
Pb	0.000000	0.000000	-2.598173
Pb	1.910992	-2.570283	-1.597852
Pb	1.910992	-2.570283	1.597852
Pb	1.910992	2.570283	-1.597852
Pb	1.910992	2.570283	1.597852
Pb	3.621362	-0.000000	2.576613
Pb	3.621362	0.000000	-2.576613
Pb	4.642258	-1.617941	0.000000
Pb	4.642258	1.617941	-0.000000

***D*$_{2h}$-[Fe$_4$Pb$_{18}$]$^{4-}$ (^{9}B$_{2u}$) PBE**; S = 4; Bond Energy = -106.1882 eV

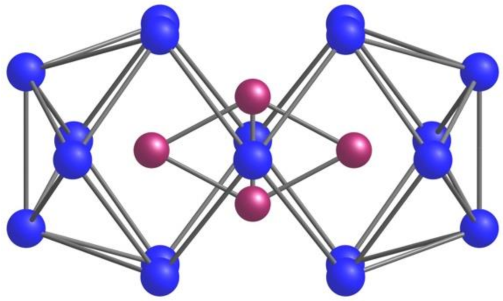

Fe	-2.082254	0.000000	0.000000
Fe	-0.000000	-1.104596	0.000000
Fe	0.000000	1.104596	0.000000
Fe	2.082254	-0.000000	0.000000

Pb	-4.663880	-1.612551	0.000000
Pb	-4.663880	1.612551	0.000000
Pb	-3.695469	-0.000000	-2.610716
Pb	-3.695469	-0.000000	2.610716
Pb	-1.908389	-2.483756	-1.603867
Pb	-1.908389	-2.483756	1.603867
Pb	-1.908389	2.483756	-1.603867
Pb	-1.908389	2.483756	1.603867
Pb	0.000000	-0.000000	2.697926
Pb	0.000000	0.000000	-2.697926
Pb	1.908389	-2.483756	-1.603867
Pb	1.908389	-2.483756	1.603867
Pb	1.908389	2.483756	-1.603867
Pb	1.908389	2.483756	1.603867
Pb	3.695469	0.000000	-2.610716
Pb	3.695469	0.000000	2.610716
Pb	4.663880	-1.612551	0.000000
Pb	4.663880	1.612551	0.000000

***D_{2h}*-[Fe_4Sn_{18}]$^{4-}$ ($^{13}B_{3g}$) PBE**; S = 6; Bond Energy = -106.0472 eV

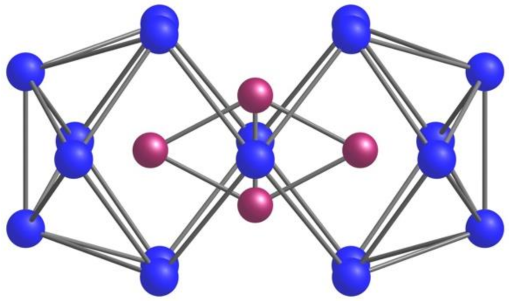

Fe	-2.172123	0.000000	0.000000
Fe	-0.000000	1.135559	0.000000
Fe	0.000000	-1.135559	0.000000
Fe	2.172123	-0.000000	0.000000
Pb	-4.753193	-1.624579	0.000000
Pb	-4.753193	1.624579	0.000000
Pb	-3.746434	-0.000000	-2.586399
Pb	-3.746434	-0.000000	2.586399
Pb	-1.983773	-2.505136	-1.599545
Pb	-1.983773	-2.505136	1.599545
Pb	-1.983773	2.505136	-1.599545
Pb	-1.983773	2.505136	1.599545
Pb	0.000000	-0.000000	2.612914
Pb	0.000000	0.000000	-2.612914
Pb	1.983773	-2.505136	-1.599545
Pb	1.983773	-2.505136	1.599545

Pb	1.983773	2.505136	-1.599545
Pb	1.983773	2.505136	1.599545
Pb	3.746434	0.000000	-2.586399
Pb	3.746434	0.000000	2.586399
Pb	4.753193	-1.624579	0.000000
Pb	4.753193	1.624579	0.000000

***D_{2h}*-$[Fe_4Sn_{18}]^{4-}$ ($^{11}B_{1g}$) PBE0**; S = 5; Bond Energy = -149.3374 eV

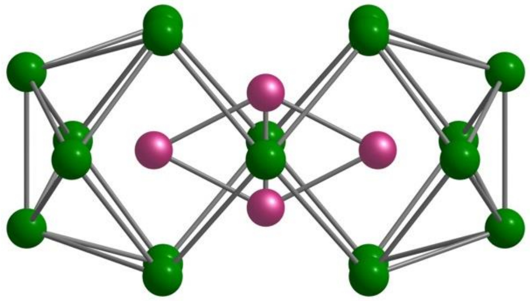

Fe	-2.174304	-0.000000	0.000000
Fe	-0.000000	-1.117717	0.000000
Fe	0.000000	1.117717	0.000000
Fe	2.174304	0.000000	0.000000
Sn	-4.643255	-1.521250	0.000000
Sn	-4.643255	1.521250	0.000000
Sn	-3.758201	-0.000000	-2.510523
Sn	-3.758201	-0.000000	2.510523
Sn	-2.000454	-2.333553	-1.569163
Sn	-2.000454	-2.333553	1.569163
Sn	-2.000454	2.333553	-1.569163
Sn	-2.000454	2.333553	1.569163
Sn	0.000000	-0.000000	2.535595
Sn	0.000000	0.000000	-2.535595
Sn	2.000454	-2.333553	-1.569163
Sn	2.000454	-2.333553	1.569163
Sn	2.000454	2.333553	-1.569163
Sn	2.000454	2.333553	1.569163
Sn	3.758201	0.000000	-2.510523
Sn	3.758201	0.000000	2.510523
Sn	4.643255	-1.521250	0.000000
Sn	4.643255	1.521250	0.000000

***D_{2h}*-$[Fe_4Sn_{18}]^{4-}$ ($^{11}B_{3g}$) PBE0**; S = 5; Bond Energy = -149.6323 eV

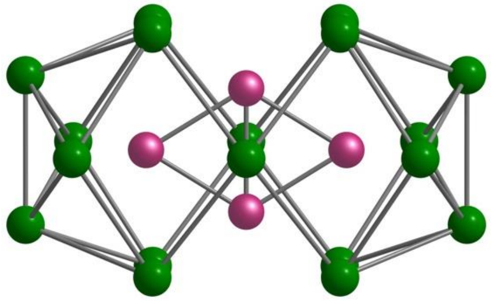

Fe	-2.040166	-0.000000	0.000000
Fe	-0.000000	-1.259874	0.000000
Fe	0.000000	1.259874	0.000000
Fe	2.040166	0.000000	0.000000
Sn	-4.492865	-1.527314	0.000000
Sn	-4.492865	1.527314	0.000000
Sn	-3.533741	0.000000	-2.483912
Sn	-3.533741	0.000000	2.483912
Sn	-1.907377	-2.484114	-1.548424
Sn	-1.907377	-2.484114	1.548424
Sn	-1.907377	2.484114	-1.548424
Sn	-1.907377	2.484114	1.548424
Sn	0.000000	-0.000000	-2.462836
Sn	0.000000	0.000000	2.462836
Sn	1.907377	-2.484114	-1.548424
Sn	1.907377	-2.484114	1.548424
Sn	1.907377	2.484114	-1.548424
Sn	1.907377	2.484114	1.548424
Sn	3.533741	-0.000000	-2.483912
Sn	3.533741	-0.000000	2.483912
Sn	4.492865	-1.527314	0.000000
Sn	4.492865	1.527314	0.000000

The optimized cartesian coordinates of singlet $[Cu_4Sn_{18}]^{4-}$ and $[Cu_4Pb_{18}]^{4-}$, with corresponding bond energies. Stationary points were confirmed.

***D_{2h}*-$[Cu_4Sn_{18}]^{4-}$ (^1A_g)**; S = 0; Bond Energy = -99.1218 eV

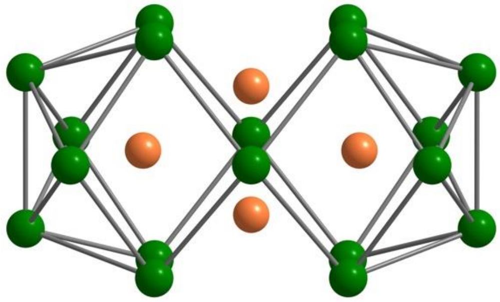

Cu	-2.170049	0.000000	0.000000

Cu	0.000000	-1.287470	0.000000
Cu	0.000000	1.287470	0.000000
Cu	2.170049	0.000000	0.000000
Sn	-4.544207	-1.593457	0.000000
Sn	-4.544207	1.593457	0.000000
Sn	-3.598326	0.000000	-2.479774
Sn	-3.598326	0.000000	2.479774
Sn	-1.926917	-2.439850	-1.523581
Sn	-1.926917	-2.439850	1.523581
Sn	-1.926917	2.439850	-1.523581
Sn	-1.926917	2.439850	1.523581
Sn	0.000000	0.000000	-2.442321
Sn	0.000000	0.000000	2.442321
Sn	1.926917	-2.439850	-1.523581
Sn	1.926917	-2.439850	1.523581
Sn	1.926917	2.439850	-1.523581
Sn	1.926917	2.439850	1.523581
Sn	3.598326	0.000000	-2.479774
Sn	3.598326	0.000000	2.479774
Sn	4.544207	-1.593457	0.000000
Sn	4.544207	1.593457	0.000000

***D_{2h}*-[Cu_4Pb_{18}]$^{4-}$ (^1A_g)**; S = 0; Bond Energy = -94.1964 eV

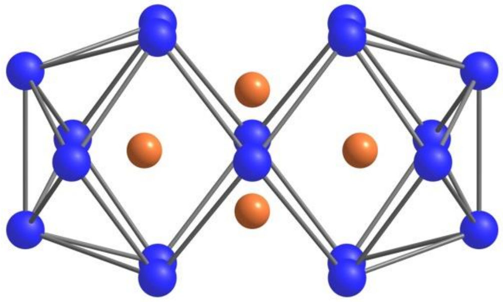

Cu	-2.217917	0.000000	0.000000
Cu	0.000000	-1.261034	0.000000
Cu	0.000000	1.261034	0.000000
Cu	2.217917	0.000000	0.000000
Pb	-4.695226	-1.660138	0.000000
Pb	-4.695226	1.660138	0.000000
Pb	-3.692693	0.000000	-2.599921
Pb	-3.692693	0.000000	2.599921
Pb	-1.940274	-2.537059	-1.592346
Pb	-1.940274	-2.537059	1.592346
Pb	-1.940274	2.537059	-1.592346
Pb	-1.940274	2.537059	1.592346
Pb	0.000000	0.000000	-2.585181

Pb	0.000000	0.000000	2.585181
Pb	1.940274	-2.537059	-1.592346
Pb	1.940274	-2.537059	1.592346
Pb	1.940274	2.537059	-1.592346
Pb	1.940274	2.537059	1.592346
Pb	3.692693	0.000000	-2.599921
Pb	3.692693	0.000000	2.599921
Pb	4.695226	-1.660138	0.000000
Pb	4.695226	1.660138	0.000000

The optimized cartesian coordinates of $[Pd_2Sn_{18}]^{4-}$, $[Fe_3Sn_{18}]^{4-}$, and $[Cd(NiSn_9)_2]^{6-}$, with corresponding bond energies. Stationary points were confirmed.

***D_{3d}*-$[Pd_2Sn_{18}]^{4-}$**; S = 0; Bond Energy = -91.7270 eV

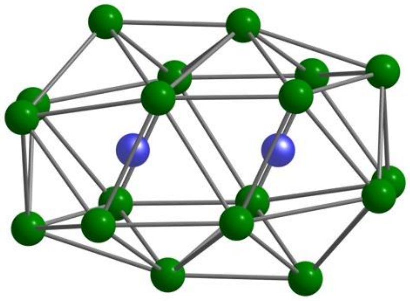

Pd	-1.630976	0.000000	-0.000000
Pd	1.630976	-0.000000	0.000000
Sn	-3.990038	-1.761383	0.000000
Sn	-3.990038	0.880692	-1.525403
Sn	-3.990038	0.880692	1.525403
Sn	-2.221834	-1.442317	-2.498166
Sn	-2.221834	-1.442317	2.498166
Sn	-2.221834	2.884633	0.000000
Sn	-0.866525	-2.802840	0.000000
Sn	-0.866525	1.401420	-2.427331
Sn	-0.866525	1.401420	2.427331
Sn	0.866525	-1.401420	-2.427331
Sn	0.866525	-1.401420	2.427331
Sn	0.866525	2.802840	-0.000000
Sn	2.221834	-2.884633	-0.000000
Sn	2.221834	1.442317	-2.498166
Sn	2.221834	1.442317	2.498166
Sn	3.990038	-0.880692	-1.525403
Sn	3.990038	-0.880692	1.525403
Sn	3.990038	1.761383	-0.000000

***D_{3d}*-$[Fe_3Sn_{18}]^{4-}$**; S = 3; Bonding Energy = -110.8432 eV

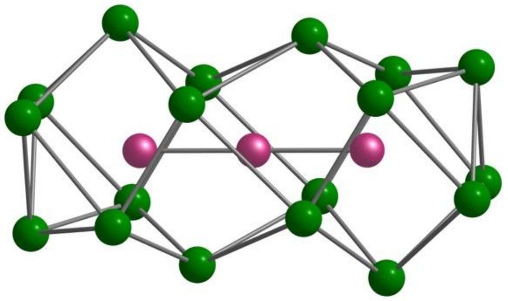

Fe	-2.416679	0.000000	-0.000000
Fe	0.000000	0.000000	0.000000
Fe	2.416679	-0.000000	0.000000
Sn	-4.759765	-1.758692	-0.000000
Sn	-4.759765	0.879346	-1.523072
Sn	-4.759765	0.879346	1.523072
Sn	-2.801062	-1.364018	-2.362549
Sn	-2.801062	-1.364018	2.362549
Sn	-2.801062	2.728036	0.000000
Sn	-1.223809	-2.438309	-0.000000
Sn	-1.223809	1.219155	-2.111638
Sn	-1.223809	1.219155	2.111638
Sn	1.223809	-1.219155	-2.111638
Sn	1.223809	-1.219155	2.111638
Sn	1.223809	2.438309	0.000000
Sn	2.801062	-2.728036	-0.000000
Sn	2.801062	1.364018	-2.362549
Sn	2.801062	1.364018	2.362549
Sn	4.759765	-0.879346	-1.523072
Sn	4.759765	-0.879346	1.523072
Sn	4.759765	1.758692	0.000000

***D_{3d}*-[Cd(NiSn$_9$)$_2$]$^{6-}$**; S = 0; Bonding Energy = -101.3659 eV

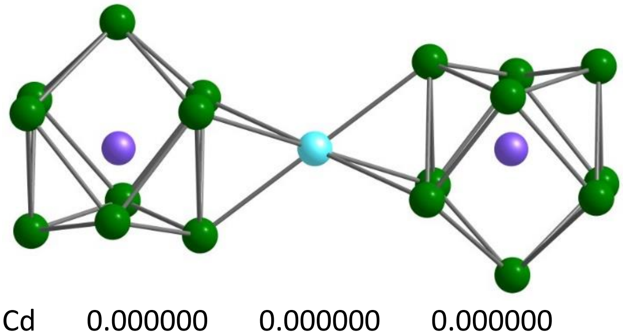

Cd	0.000000	0.000000	0.000000
Ni	-4.268052	-0.000000	0.000000
Ni	4.268052	0.000000	-0.000000
Sn	-6.171905	-0.903532	-1.564964
Sn	-6.171905	-0.903532	1.564964

Sn	-6.171905	1.807064	0.000000
Sn	-4.290071	-2.743711	0.000000
Sn	-4.290071	1.371856	-2.376124
Sn	-4.290071	1.371856	2.376124
Sn	-2.501178	-0.948112	1.642179
Sn	-2.501178	-0.948112	-1.642179
Sn	-2.501178	1.896225	-0.000000
Sn	2.501178	-1.896225	0.000000
Sn	2.501178	0.948112	-1.642179
Sn	2.501178	0.948112	1.642179
Sn	4.290071	-1.371856	-2.376124
Sn	4.290071	-1.371856	2.376124
Sn	4.290071	2.743711	-0.000000
Sn	6.171905	-1.807064	-0.000000
Sn	6.171905	0.903532	-1.564964
Sn	6.171905	0.903532	1.564964

7. References

[1] G. Sheldrick, *Acta. Cryst. A.*, 2015, **71**, 3-8.

[2] O. V. Dolomanov, L. J. Bourhis, R. J. Gildea, J. A. K. Howard, H. Puschmann, *J. Appl. Crystallogr.* **42**, 339-341 (2009).

[3] A. Spek, *Acta. Cryst. D.*, 2009, **65**, 148-155.

[4] A. Spek, *Acta. Cryst. C.*, 2015, **71**, 9-18.

[5] J. P. Perdew, K. Burke, M. Ernzerhof, *Phys. Rev. Lett.*, 1996, **77**, 3865-3868.

[6] M. Ernzerhof, G. E. Scuseria, *J. Chem. Phys.*, 1999, **110**, 5029-5036.

[7] S. Grimme, *J. Comput. Chem.*, 2004, **25**, 1463-1473.

[8] Z.-S. Li, W.-X. Chen, H. W. T. Morgan, C.-C. Shu, J. E. McGrady and Z.-M. Sun, *Chem. Sci.*, 2024, **15**, 1018-1026.

[9] Y.-N. Yang, L. Qiao and Z.-M. Sun, *Chin. Chem. Lett.*, 2023, **34**, 107207.

[10] Z.-M. Sun, H. Xiao, J. Li and L.-S. Wang, *J. Am. Chem. Soc.,* 2007, **129**, 9560-9561.

[11] F. K. Sheong, W.-J. Chen, J.-X. Zhang, Y. Li and Z. Lin, *Dalton Trans.*, 2017, **46**, 2214–2219.

[12] J.-X. Zhang, F. K. Sheong and Z. Lin, *Inorg. Chem.*, 2019, **58**, 3473–3478.

[13] J.-X. Zhang, F. K. Sheong and Z. Lin, *WIREs Comp. Mol. Sci.*, 2020, **10**, e1469.

[14] L. Qiao, C. Zhang, C.-C. Shu, H. W. T. Morgan, J. E. McGrady and Z.-M. Sun, *J. Am. Chem. Soc.*, 2020, **142**, 13288–13293.
